# Health risk assessment of heavy metals in arid soils of the Nile Delta, Egypt using GIS and multivariate statistical analyses

**DOI:** 10.1371/journal.pone.0335394

**Published:** 2025-11-10

**Authors:** Ehab Hendawy, Abdel-Aziz Belal, Nazih Y. Rebouh, Mohamed S. Shokr, Abd El Aziz S. Sheta, Ahmed M. Saleh, Ayman F. Abou-Hadid

**Affiliations:** 1 National Authority for Remote Sensing and Space Sciences, Cairo, Egypt; 2 Institute of Postgraduate Studies and Agricultural Research in Arid Regions, Ain shams University, Cairo, Egypt; 3 Institute of Environmental Engineering, RUDN University, Moscow, Russia; 4 Soil and Water Department, Faculty of Agriculture, Tanta University, Tanta, Egypt; 5 Soil Science Department, Faculty of Agriculture, Ain Shams University, Cairo, Egypt; 6 Horticulture Department, Faculty of Agriculture, Ain shams University, Cairo, Egypt; Makerere University College of Natural Sciences, UGANDA

## Abstract

Economic expansion has led to an increase in new toxins in the environment, creating a global problem for managing both environmental and human health. This study aimed to quantify the concentration of heavy metals (HMs) in soils of the Kafr El-Sheikh Governorate, located in the northern Nile Delta, Egypt, and to evaluate potential health risks by integrating Geographic Information Systems (GIS) with multivariate statistical analyses. Soil samples from 27 sites were analysed for potentially toxic elements (As, Cd, Co, Cu, Fe, Mn, Ni, Pb, and Zn) using Inductively Coupled Plasma Mass Spectrometry. Soil pollution indices such as enrichment factor (EF), contamination factor (CF), and geoaccumulation index (I_geo_) were assessed. In addition, non-carcinogenic and cancer risk indices were calculated. To identify the origins of HMs in the research area, Pearson’s bivariate correlation, principal component, and hierarchical cluster analyses (PCA) were used. The findings revealed that the mean HM concentrations (mg kg^-1^) were in the following order: Fe (10706 ± 2855)>Mn (697.53 ± 138.46)> As(210.07 ± 20.23)> Zn (207.40 ± 216.76)>Ni(112.43 ± 13.68)> Cu (87.15 ± 47.69)> Pb(31.11 ± 8.66)> Co(23.97 ± 5.96)> Cd (6.50 ± 5.62). The EF, CF, and Igeo indices indicated that the soils in the study area were contaminated with metals. The risk index values indicated moderate, considerable, and very high ecological risk, with a median value of 2060.40 (range: 192.95–5006.97). From PCA results, the possible sources of the metals in the arid soils included pesticides and chemical fertilizers, except for Mn, which appeared to originate from geogenic sources. Both children and adults had hazard quotient and Hazard index values less than one in all three exposure pathways, except for As in the ingestion pathway for children.. Furthermore, the total cancer risk (sum of ingestion, inhalation, and dermal contact pathways for each element) associated with children’s exposure to the elements under investigation was as follows: Pb (4.5E-02)> As (4.1E-03)> Ni (2.6E-03)> Cd (4.7E-05). Consequently, the largest cancer risk was determined to be from Pb. These results provide valuable information that emphasizes the need to mitigate pollution from potentially toxic elements in the Nile delta and minimize health concerns for the local population.

## 1. Introduction

The quality of soil has a significant impact on food safety, crop product quality, and ultimately human health [1,2]. Soil is a complex, living, ever-changing, and dynamic component of the ecosystem that is crucial to human survival and societal progress. The environment and soil have been contaminated in recent decades by the fast growth of industrialization and urbanization [3–5]. They are employed in many commercial and industrial fields, including electronics, transportation, and construction [6]. The need for HMs has grown over time as the world’s inhabitants and economies continue to grow. Egypt’s Nile Delta is home to a sizable agricultural region and is an important economic sector. The ongoing industrialization and urbanization of the Nile Delta and its environs have led to increased contamination of soils and water supplies, which poses a potential health risk [7,8]. Egypt’s rapidly rising soil contamination has become a serious threat to both the economy and public health. The production of crops on Egyptian land was moved to multiple seasons of the year. To boost crop output and lower crop losses, they thus utilize enormous amounts of mineral fertilizers and pesticides without observing any restrictions, especially in the Nile Delta. [9]. Over the past few years, a number of studies on the ecological danger to soils in various regions of Egypt and trace metal pollution of agrarian soil have been conducted. Wastewater from industrial and agricultural sources that discharge into the Nile water contains a lot of contaminants. Accordingly, over time, the contamination caused by trace metals that are transmitted from the water to the soil has increased [10–12]. In these kinds of areas, farmers occasionally rely on drainage water to irrigate their soils [13,14]. These suggest that human activity may have an impact on agricultural soil pollution, which needs further study in the future.

Heavy metals can have a detrimental effect on human health in addition to the environment [15]. They can build up in the human body, resulting from extended exposure to their effects. In this manner, several kinds of diseases emerge [16]. Acute and chronic illnesses affecting the immunological, neurological, cardiovascular, endocrine, skeletal, and other systems are caused by the buildup of HMs in organs [17]. HMs are present in humans in a variety of ways, ingestion, inhalation, or direct skin contact are the initial ways they come into contact with the soil. Another method is indirectly through food produced on tainted soil. Determining the level of pollution in agricultural soil is crucial to guarantee the safety of food [18,19]. The quality of crops is eventually threatened by soil pollution brought on by industry and agriculture because HMs are harmful to human health when consumed through contaminated food [20,21]. Heavy metals contain elements that are harmful at low concentrations, including As, Cd, Hg, and Pb, as well as elements that are essential for mammals, like Cr, Cu, Ni, and Zn. However, even necessary HMs can be extremely harmful and cause major health issues if they get into the body in high enough amounts [22–25]. Long-term exposure to HMs is linked to an increased risk of getting cancer [26], and HMs can cause damage to a variety of organs [27]. HMs interfere with the events occurring within the cells by altering their redox potential once they get there [23]. To give a thorough description of the state of HMs contamination in soil, pollution indices have been used extensively and effectively. Shokr et al,. [28] observed that majority of soil samples from the central part of the Nile Delta had pollution load indices > 1, indicating significant to high polluted classes with HMs. Omran [29] realized that the soil of the Bahr El Baqar area had moderate to very high levels of HMs. Khalifa and Gad [12] noticed that the soil of the Quessna district in the Southwest Nile Delta had the highest levels of pollution and potential ecological risk. Knowing the causes of contamination and comprehending the geographical distribution of heavy metals are the first steps towards properly managing soil pollution [30]. Thus, mapping the spatial distribution of soil characteristics is facilitated by geographic information systems (GIS) [31–34]. Spatial data can be studied, and where the unsampled data is located can be anticipated using a technique known as geostatistical analysis [35]. Numerous techniques, such as the index approach, quotient method, fuzzy comprehensive assessment, geoaccumulation index, prospective ecological risk index, and pollutant load index, are used to evaluate soil ecological risk [4,36]. The Nile delta region uses wastewater for irrigation; the main drain in El Gharbia (Kitchener), Egypt, mixes Nile water with wastewater from industry and agriculture [37].It is essential to track the level of HM contamination in the soil to estimate the possible health risk, control these pollutants effectively, and ensure food safety [32]. In the context of arid and semi-arid environments, few studies have combined geospatial techniques (GIS) with geochemical analysis and health risk assessment, despite the fact that several studies have examined soil contamination and its effects on the environment. The spatial distribution of contaminants or the geochemical background of soils has been the primary subject of previous research, but these have rarely been integrated with a methodical assessment of possible hazards to human health. Therefore, this study’s three primary goals were to: (1) identify the concentrations, sources, and spatial distributions of heavy metals in the North Nile Delta (2) evaluate the soil’s heavy metal pollution; and (3) assess the heavy metals’ possible health risks based on different exposure routes, to assist local governments in successfully preventing and controlling the contamination of soil by metals (loids).

## 2. Methodology

### 2.1. Research area description

The study area is situated within Kafr El-Sheikh Governorate, the north Nile delta, Egypt, encompassing a region around the Kitchener Drain with a total area of 562.45 km² (56,245 hectares). The Gharbia Main Drain, commonly known as the Kitchener Drain, is among the largest and most significant drainage systems in the Nile Delta. Centrally located in the Middle Nile Delta, this drainage system spans approximately 69 km², originating in the El-Gharbia Governorate and extending northward through Kafr El-Sheikh Governorate before emptying into the Mediterranean Sea. Within the research area, the Kitchener Drain covers a length of 60.4 km².

**Fig 1:**
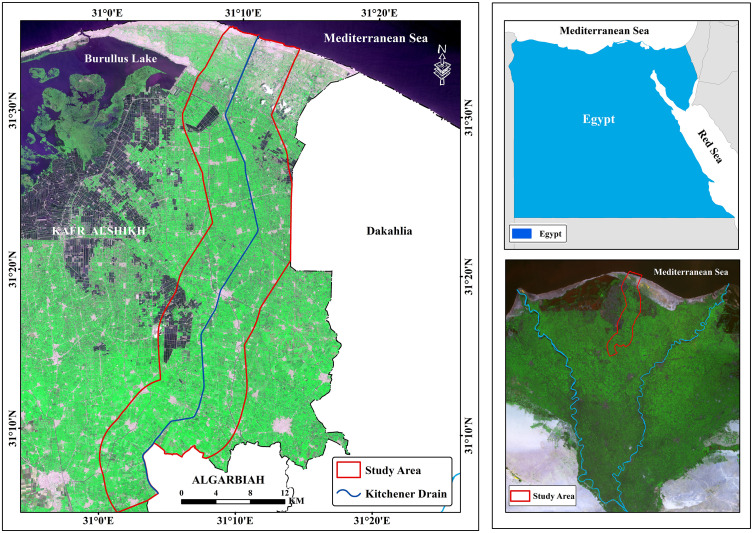
Research area location.

Geographically, the study area lies between the coordinates 31° 0’ 1.376“ to 31° 13’ 44.618” E longitude and 31° 4’ 47.090” to 31° 35’ 30.654” N latitude (Fig 1). The satellite image was downloaded from https://browser.dataspace.copernicus.eu/?zoom=5&lat=50.16282&lng=20.78613&demSource3D=%22MAPZEN%22&cloudCoverage=30&dateMode=SINGLE accessed on (9^th^ October 2024). Special permits were not required for field site access or research activities because the study was conducted on publicly accessible property and did not include interactions with regulated species or ecosystems.

**Fig 2:**
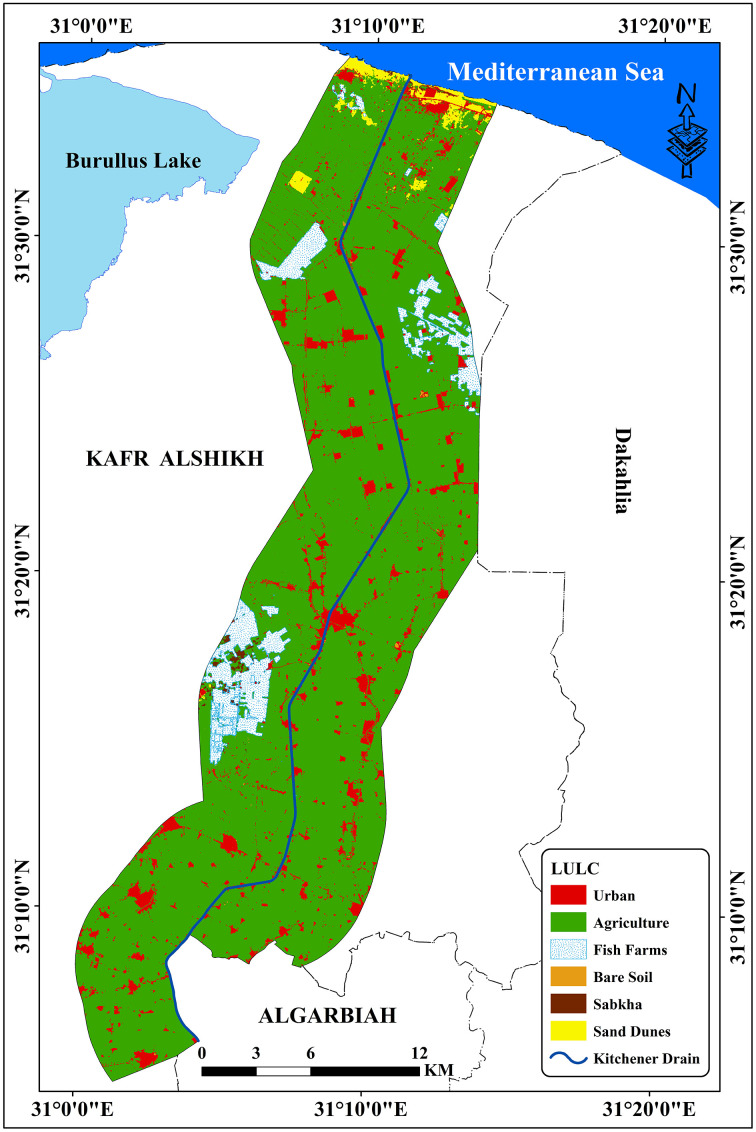
LULC of the study area after Hendawy [34].

**Fig 3:**
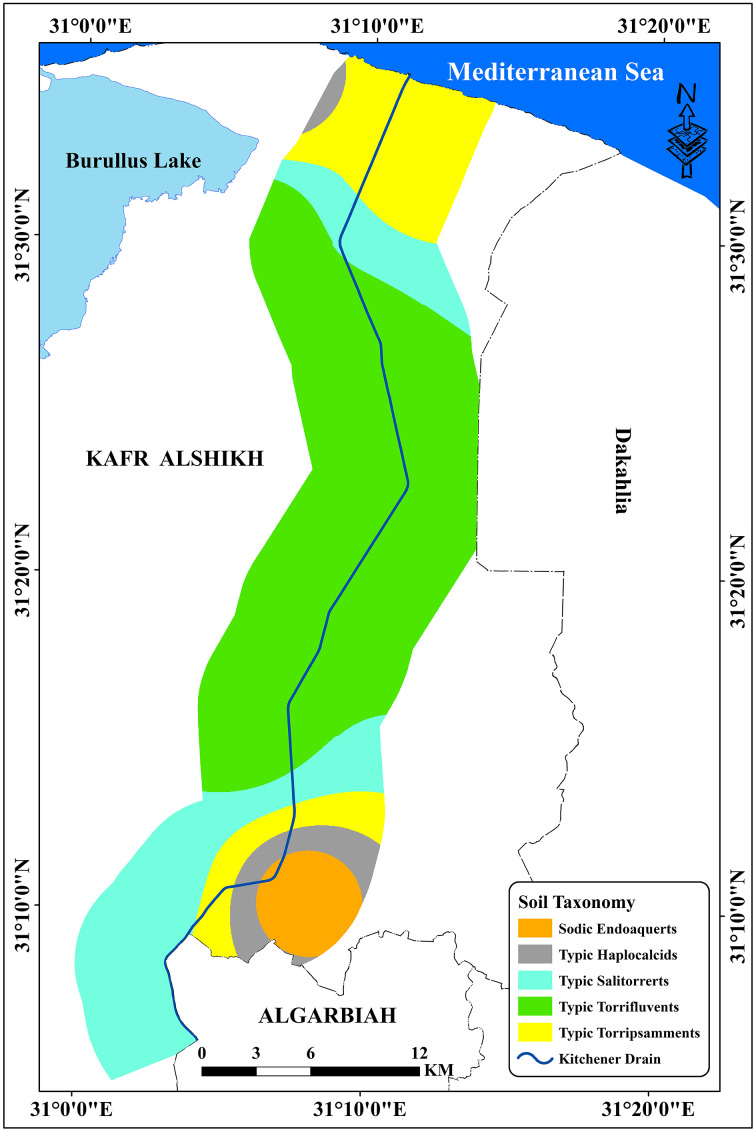
Soil taxonomy of the study area.

**Fig 4:**
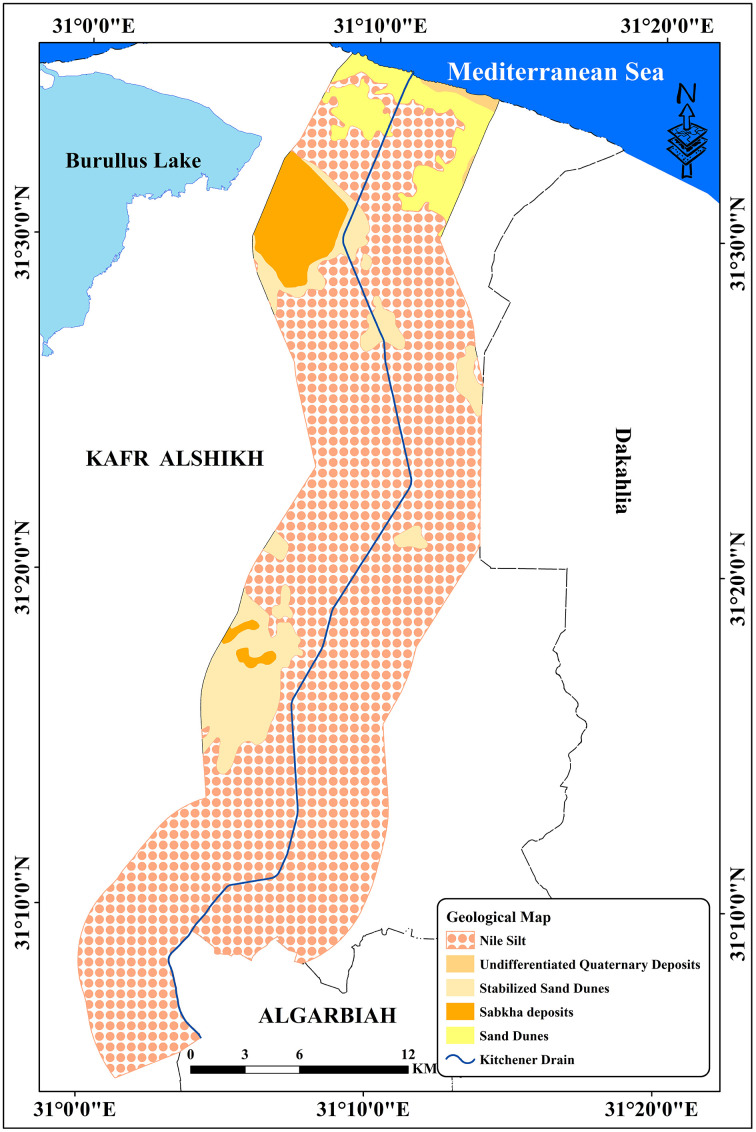
Geological map of the study area.

The region’s strategic location highlights its importance for agricultural drainage and environmental management within the Nile Delta. Additionally, the Kitchener Drain plays a critical role in supporting the region’s water management system, which significantly impacts agricultural productivity and ecological balance. Detailed examination of this area provides valuable insights into the interplay of drainage infrastructure and land use dynamics in the central Nile Delta. The research area included six classes: Agriculture, urban, fish farms, sabkha, sand dunes, and bare soils [34]. Agricultural areas account for around 91.14% of the total area, including urban regions with industrial zones, and residential sectors, which make up for 4.61% of the area under study [34] (Table 1, and Fig 2). Due to the lack of clean irrigation water, some farmers irrigate their crops with Kitchener drain water, which is why it is considered a pollutant [37]. Table 2 and Fig 3 showed the spatial distribution of different soil types in the examined area. *Typic Torrifluvents* is the most widespread soil type, according to the data, with a substantial area of 296.60 km². *Typic Salorthids*, which cover 128.11 km^2^, come next, with 88.20 km^2^, *Typic Torripsamments* are the third most common form. At 26.06 km² and 23.48 km², respectively, *Sodic Endosquerts and Typic Haplocalcids* are the smallest areas in comparison. This distribution emphasizes how saline and arid soil orders, such as Torrifluvents and Salorthids, are prevalent in dryland areas [38,39]. According to structural analysis, the Nile Delta region has been tectonically regulated from the late Eocene and Oligocene, when it was exposed to tectonic uplift. Eocene by the NW-SE and ENE-WSW fault systems, which trend parallel to the Gulf of Suez Red Sea and the Mediterranean Sea, respectively [40]. The Nile Delta can be separated into three zones, namely the southern, middle, and northern zones [41]. Coarse Nile sediments, mostly sand deposits, define the southern zone. The middle zone is thought to be a transitional zone between the southern and northern Delta zones since it is often distinguished by finer sediments than the southern zone. The terrain of the middle Delta generally slopes from east to west [1], making the level of the Damietta branch higher than that the Rosetta branch by two meters. Of the three zones, the northern zone has the finest neonile sediments. Several brackish lagoons (Maryut, Idku, Burullus, and Manzala) that are connected to the Mediterranean Sea by slender outflows define the northern portion of the Delta. The distribution of the various geological units in the research region is shown in Table 3 and Fig 4. At roughly 457.16 km^2^, the Nile Silt has the greatest coverage, followed by Stabilized Sand Dunes at 48.87 km². The Sabhka deposits occupy around 25.73 km², whilst the Sand Dunes comprise about 28.50 km². Lastly, with just 2.19 km^2^, the Undifferentiated Quaternary Deposits represent the smallest area. These findings demonstrate the region’s geological diversity, with the Nile Silt standing out as the most prevalent unit in relation to the others [42].

**Table 1:** Land use and land cover (LULC) distribution across the study region.

LULC	Area km^2^	%
Agriculture	899.91	91.14
Urban	45.56	4.61
Fish Farms	31.67	3.21
Sabkha	1.63	0.16
Sand Dunes	7.82	0.79
Bare Soil	0.75	0.08

**Table 2:** Soil types of the investigated area.

Soil types	Area (km^2^)
*Typic Torrifluvents*	296.60
*Typic Salitorrerts*	128.11
*Typic Torripsamments*	88.20
*Sodic Endoaquerts*	26.06
*Typic Haplocalcids*	23.48

**Table 3:** Geological units of the study area.

Geological units	Area (km^2^)
Nile Silt	457.16
Sabkha deposits	25.73
Sand Dunes	28.50
Stabilized Sand Dunes	48.87
Undifferentiated Quaternary Deposits.	2.19

### 2.2. Extracting landform units

We used Arc Scene’s 3D V10.8.1. visualisation mode to extract landform units from a multi-spectral Sentinel-2 image (https://www.copernicus.eu/en/access-data/conventional-data-access-hubsaccess on October 2024), and DEM. This method revealed elevation differences in each landscape. We were able to separate landform units based on the visual interpretation of the satellite image and DEM. We also conducted a field check using previous studies [28,43,44].

### 2.3. Sampling and sample preparation

**Fig 5:**
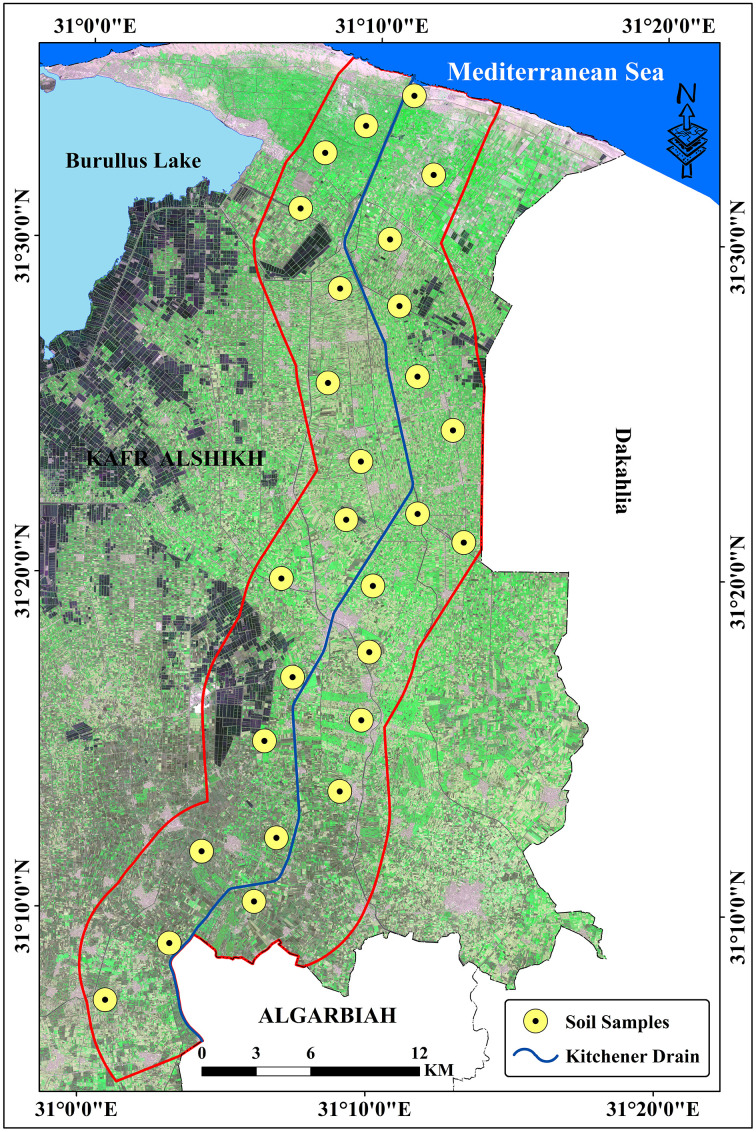
Soil samples distribution in research area.

A total of 27 soil samples were collected (Fig 5) to represent different geomorphological units. From each sampling site, three replicate samples, each weighing approximately one kilogram, were combined to create a single composite sample. These composite samples were securely stored in plastic bags and transported to the laboratory for analysis. Upon arrival, the samples were air-dried, and ground to pass through a 2-mm sieve.

The total concentrations of heavy metals, including As, Cd, Co, Cu, Fe, Mn, Ni, Pb, and Zn, were extracted following the USEPA Method 3052 for microwave-assisted acid digestion. The procedure utilized a mixture of concentrated nitric acid (HNO₃), hydrochloric acid (HCl), and hydrofluoric acid (HF), as recommended by Schumacher [45]. Specifically, a 0.50 g soil sample was placed in a 100-mL Teflon microwave digestion vessel with 5.0 mL of HNO₃ (16 M), 2.0 mL of HCl (12 M), and 1.0 mL of HF (29 M). The vessels were then heated to 180°C using a microwave digestion system (Mars-X, HP-500 Plus, CEM Corporation) until digestion was complete. After cooling, the digests were transferred to 50-mL volumetric flasks, diluted with deionized water, and stored for subsequent analysis.

### 2.4. Analysis of soils

Heavy metal concentrations were quantified using Inductively Coupled Plasma Mass Spectrometry (ICP-MS; Thermo iCAP‐RQ, USA) in NARSS. The analytical performance of the ICP-MS instrument was thoroughly evaluated by determining the limits of detection (LODs), limits of quantification (LOQs), and linearity. Linear regression analysis was used to calculate the correlation coefficients (R²=>0.990) for each metal, confirming the accuracy and reliability of the instrument (S1 Table in S1 File). To evaluate the accuracy and precision of the analytical methods used for the multi-element evaluation of soil samples, a Certified Reference Material (CRM) was examined. Containing a reagent blank to measure the background and certified reference materials from Western Australia (CRM11:EMOG17) to ensure data precision before release (S2 Table). The precision of the employed methodology is reliably indicated by recoveries with a range of 95.23% to 100%.

### 2.5. Soil pollution assessment

Enrichment factor, geo-accumulation index, and contamination factor were used to evaluate the pollution indices of nine heavy metals in the soils from the northern Nile Delta [46,47].

#### 2.5.1. Enrichment factor.

Measured metal standardisation against a reference metal serves as the basis for the enrichment factor (EF) of HMs. Fe was selected as the normalisation metal, and the reference metal needs to be naturally occurring in the research region [48]. Using Fe as the reference, the EF was computed using equation (1) below:


EF=(M÷Fe)sample/(M÷FE)background (1)

Where the ratio of each metal’s concentration to the soil sample’s iron concentration is called the (M÷Fe) sample, and the ratio of the trace metal background value to the iron background value is called the (M÷Fe) background. Based on the EF values, the soil contamination was divided into six categories, which are shown in S3 Table [49].

#### 2.5.2. Geoaccumulation index (I_geo_).

Utilizing equation (2) provided by Muller [50], the I_geo_ was computed to estimate the metal load enrichment in the soil above the baseline level.


$$I_{geo}=\;{\text{log}}_{\text{2}}\left[\frac{Cn}{\text{1}.\text{5}Bn}\right]$$ (2)

Where Bn represents the heavy metal background value and Cn represents the soil’s heavy metal concentration. The background value is determined by the average upper earth crust, according to Wedepohl [51]. According to Stoffers et al., [52] the constant 1.5 was used to account for the likely natural variations in background values that could be ascribed to different lithologic causes. According to Muller [50], the I_geo_ value can be divided into seven groups (S3 Table).

#### 2.5.3. Contamination factor.

The current study uses the contamination factor to determine the soil’s contamination status. The following formula 3 is used to calculate the factor of contamination.


CF=Cm /Cn (3)

In soil samples, Cm represents the average measured concentration of a metal, while Cn represents the metal’s value in background values. The categorization of CF is shown in Table S3 [46].

#### 2.5.4. Ecological risk index (ER).

The possible environmental risk of heavy metal deposition in soil was assessed using the indicator of ecological risk (ER). The characteristics of heavy metals and how they behave in soil were taken into account while evaluating the possible ecological impact of heavy metals [53]. The evaluation may additionally consider the environmental links with heavy metals, pollution levels, and the synergistic effects of several elements or each given substance. The ecological risk index (equation 4) is defined as follows:


ERI=CF×TR (4)

Where the literature [54–57], provides the heavy metal toxicity coefficient (Tr). The adopted Tr coefficients for Ni, Mn, Cu, Zn, Pb, Cd, and Co are 5,1,5,1,5, 30, and 5, respectively. One way to quantitatively quantify the possible environmental harm of the metals under consideration is through the indicator of ecological risk (ER) [58]. S4 Table shows the ecological risk classification based on heavy metal ER index values.

#### 2.5.6. Potential ecological risk index (RI).

The value of ER for each heavy metal under consideration was added up to create the aggregated potential ecological risk index (RI) [53]. The sum of the factors taken into consideration is known as the potential ecological risk index (equation 5). S4 Table lists the possible ecological hazards associated with heavy metals.


$$RI=\sum_{i=1}^{n}{ER}_{I}$$ (5)

### 2.6. Human health risk assessment

The health risk was estimated qualitatively and quantitatively using equations 6–14. In this investigation, the proposed United States Environmental Protection Agency (USEPA [59]), health risk assessment technique was used to investigate the potential human health risks associated with HMS exposure. Human health hazards from contaminant exposure are classified into two categories: non-cancer and cancer. Non-cancer risk can be measured for both carcinogens and non-carcinogenic PTEs; however, cancer risk can only be computed for carcinogens. In this study, two age groups of children (3–12 years old) and adults (18–40 years old) from the study area were evaluated for non-cancer and cancer risks via three exposure pathways: ingestion, inhalation, and skin contact with soil metals.

The first step in determining the carcinogenic and non-carcinogenic risk is to use Equations 6–8 to determine the chronic daily intake (CDI) of heavy metals (HMs) from the soil. It serves as the foundation for subsequent computations and the ultimate risk evaluation. While most researchers consider all three exposure pathways (ingestion, inhalation, and dermal contact) [58,60–63] when evaluating the health risk, some researchers only consider ingestion [64,65] or ingestion and dermal contact [18,22,66–68], because inhalation carries the lowest risk [22,65,68]. Second, using the hazard index (HI) and hazard quotient (HQ) provided by equations 9–12, the non-cancer risk was calculated. Finally, equations 13, and 14 were used to determine the carcinogenic risk (CR) of each carcinogen element. The following formulas were used to estimate the health risk assessment:


$${CDI}_{ing=C_{soil\times }ingR\times EF\times \frac{ED}{BW\times AT}\times 10^{-6}}$$ (6)

$${CDI}_{inh=C_{soil\times }inhR\times EF\times \frac{ED}{PEF\times AT\times BW}}$$ (7)

$${CDI}_{derm=C_{soil\times }SA\times AF\times ABS\times EF\times \frac{ED}{BW\times AT}\times 10^{-6}}$$ (8)

$${HQ}_{ing={CDI}_{ing\;÷\;}{Rfd}_{ing\;\;}}$$ (9)

$${HQ}_{inh\;\;={CDI}_{iinh\;\;÷\;}{Rfd}_{inh\;\;}}$$ (10)

$${HQ}_{derm\;\;={CDI}_{derm\;\;÷\;}{Rfd}_{iderm\;\;}}$$ (11)

$$HI=\sum_{K=1}^{9}HQ$$ (12)

$${CR}_{ing/inh/derm\;\;=C_{ing/inh/derm\;\times }CSF}$$ (13)

$$Total\;carcinogenic\;risk\left(TCR\right)=\sum_{K=1}^{4}{CDI}_{ing/inh/derm}$$ (14)

Where:

Ing is the ingestion pathway, inh is inhalation, derm is dermal contact

S5 Table lists the parameters and underlying presumptions for evaluating metal(loid)s exposure through various channels. Additionally, S6 Table displays the cancer risk factors (CSFs) and reference doses (Rfds) used for health risk assessment in this study. For the purpose of this risk assessment, carcinogens are evaluated based on the CR/CRt ratio. A clear threshold is established to differentiate between negligible and significant risk levels. Carcinogens with a CR/CRt value less than 1.00E-04 are considered to pose a negligible to low risk of cancer; consequently, their risk can be overlooked. Conversely, CR/CRt levels greater than 1.00E-04 indicate a potentially high risk and are recognized as having the potential to lead to cancer, necessitating further attention and mitigation strategies [69,70].

### 2.7. Statistical analysis

The software SPSS (version 25, IBM, New York, USA) and Python (Version 3.13, Guido van Rossum, Netherlands) were used to calculate the data’s minimum, maximum, mean, standard deviation (SD), and Pearson correlation. Principal Component Analysis (PCA) was used to ascertain the relationships between the HMs and their potential sources, and its validity was evaluated by the Bartlett sphericity tests (p < 0.001) and the Kaiser–Meyer–Olkin (KMO) value (KMO > 0.5) [71]. The hierarchical cluster analysis (HCA) was applied to group the studied soil samples based on their heavy metal concentrations.

## 3. Results and discussion

### 3.1. Geomorphological map of the investigated area

**Fig 6:**
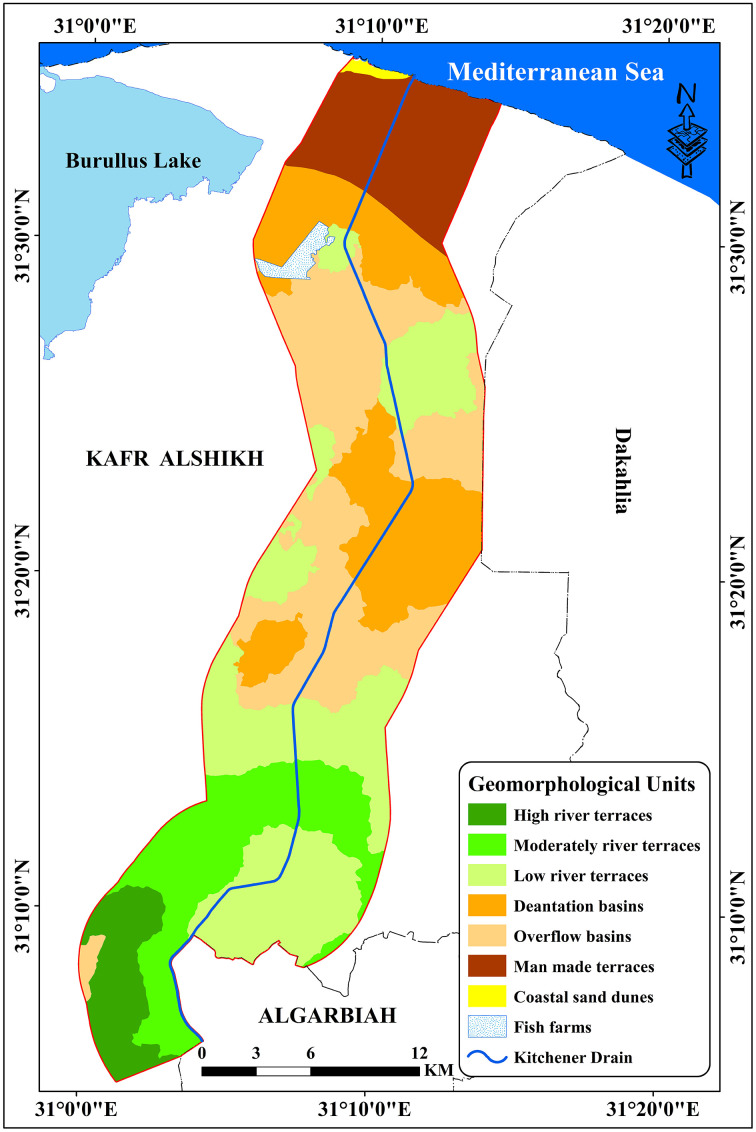
Geomorphological units in the investigated area.

The landforms in the study area were identified and characterised using the Digital Elevation Model (DEM), Sentinel-2 data, and field checks. According to the data (Table 4 and Fig 6), the principal physiographic units in the research region are the flood plain, lacustrine plain, and marine plain. The flood plain is the study region’s principal landform, accounting for 529.11 km^2^ (98.80% of the total area). The flood plain’s relief is virtually level and flat. These units are very common in the Nile delta, Egypt [72]. The units are included decantation basins (102.53 Km^2^), High River terraces (29.60 km^2^), Moderately River terraces (73.70 km^2^), low river terraces (135.82 Km^2^), man-made terraces (60.74 km^2^), and overflow basins (126.73 Km^2^). The lacustrine plain, which includes the fish farm unit, covers 4.61 km^2^ (0.86% of the total area) and is characterised by flat, nearly flat, to gently undulating relief. The marine plain, which covers 1.37 km^2^ (0.32% of the total area), is located in the north of the study area and has a topography that is low to almost flat and gradually undulating, as evidenced by the coastal plain.

**Table 4:** Geomorphological units’ area of the investigated area.

Mapping units	km^2^	%
Coastal sand dunes	1.73	0.32
Decantation basins	102.53	19.15
Fish farms	4.61	0.86
High river terraces	29.60	5.53
Low river terraces	135.82	25.36
Man-made terraces	60.74	11.34
Moderately river terraces	73.70	13.76
Overflow basins	126.73	23.67

### 3.2. Heavy metal concentration in the soils

Overall, the following pattern was seen in the mean concentrations of the HMs in the soil samples: Fe (10706 ± 2855 mg kg^-1^)> Mn (697.53 ± 138.46 mg kg^-1^)>As (210.07 ± 20.23 mg kg^-1^)>Zn (207.40 ± −216.76 mg kg^-1^)>Ni (112.43 ± 43.68 mg kg^-1^)>Cu (87.15 ± 47.69 mg kg^-1^)> Pb (31.11 ± 8.66 mg kg^-1^)> Co (23.97 ± 5.96 mg kg^-1^)> Cd (6.50 ± 5.62 mg kg^-1^) (Table 5). It is generally accepted that, As in soil is of geological origin, with clayey soils having a greater background concentration. But because arsenic released from man-made sources vastly outweighs that from natural sources, anthropogenic arsenic pollution is rather common [73]. Zinc is essential for life and serves as a structural or catalytic component of a number of enzymes that are involved in transcription, translation, and energy metabolism [74,75]. While some natural processes contribute to the entry of Zn into the air, water, and soils, human activities—such as mining, Zn purification, steel manufacture, coal burning, and waste disposal—are mostly responsible [76]. For both human and animal health, Ni is an essential trace element, particularly for the production of red blood cells, but excessive amounts can be harmful [77]. Because Ni is readily and quickly taken by plants, it may accumulation in crops [78]. Cu is one of the elements that is necessary for human health, for example, since it is a component of enzymes that are engaged in certain metabolic processes. But at higher dosages, it can be dangerous since it can damage the liver, immune system, neurological system, reproductive system, and cause gastrointestinal distress [79]. Because copper accumulation in soils is mostly caused by human activity, such as the widespread use of copper-containing agricultural products, particularly pesticides [80], soil samples with high concentrations of copper may be noticed in Mediterranean nations. Small amounts of Pb, a bluish-gray metal that occurs naturally, are present in the crust of the Earth [76]. A large portion of the lead found in the environment comes from human activities like burning fossil fuels [76]. Co is vital to human health (it is a component of vitamin B12, for example), but excessive levels of it can have detrimental effects on the heart and lungs [79]. Noteworthy is the relatively poor transmission potential from soil to plant edible sections [81]. Anthropogenic sources of Ni and Co contamination in soils include sewage sludge and other wastes used as soil conditioners, agricultural fertilisers, notably phosphates, atmospheric deposition, and inorganic fertilisers [37]. Cadmium is exceedingly toxic and serves no biological purpose. In recent decades, there has been a significant increase in cadmium-related environmental pollution due to its increasing industrial use [74]. The average amounts of Ni, Cu, and Pb exceeded the Department of Environmental Affairs’ [82] recommendations, although the remaining elements were lower (Table 5). Except for Mn, the means of all heavy metals studied surpassed the chemical composition of the upper continental crust as determined by Taylor and McLennan [83] and the natural concentration of heavy metals in rocks as calculated by Bradl [84] (Table 5).

**Table 5:** Statistics of studied variables in the study area after [34].

Sample	As	Cd	Co	Cu	Fe	Mn	Ni	Pb	Zn
Measuring units	mg kg^-1^								
Samples	228.392	12.312	24.903	217.782	11,416.57	632.43	135.648	34.281	1201.77
S 1	221.74	14.985	26.622	112.563	9,863.42	630.639	149.67	40.473	186.453
S 2	222.904	14.814	26.271	103.986	11,866.35	653.391	153.027	32.904	130.329
S 3	201.865	12.726	26.145	207.45	8,464.78	717.813	201.474	39.852	419.337
S 4	206.855	16.02	28.557	133.47	12,051.42	655.11	162.864	58.095	396.693
S 5	226.62	14.895	27.963	100.377	10,584.48	664.884	147.087	30.924	123.588
S 6	218.579	11.997	33.462	116.163	13,982.00	978.597	166.869	35.721	174.483
S 7	214.138	12.906	29.124	113.4	14,318.87	862.956	144.405	39.816	268.848
S 8	206.996	12.978	26.091	107.793	13,106.14	650.322	168.399	38.727	236.466
S 9	255.339	10.197	25.308	85.131	11,158.99	650.457	115.974	33.03	197.964
S 10	194.94	4.077	23.202	80.91	8,499.65	569.781	108.891	22.41	254.484
S 11	188.769	4.113	22.122	74.493	9,982.73	424.197	110.43	18.576	90.909
S 12	206.96	1.152	31.545	86.715	12,886.38	783.531	109.44	32.499	169.776
S 13	204.277	4.707	27.729	89.775	14,623.04	629.82	135.45	22.923	190.017
S 14	200.803	4.788	24.471	108.225	9,320.82	587.889	122.319	23.499	133.02
S 15	183.385	3.384	20.619	60.894	12,120.81	658.665	99.531	16.092	86.526
S 16	194.651	4.518	26.811	84.006	13,519.32	757.206	125.622	24.264	114.759
S 17	200.446	3.789	24.552	68.814	12,697.68	879.588	100.791	26.019	180.108
S 18	189.306	2.502	16.866	48.159	11,290.99	590.886	71.523	25.011	176.967
S 19	189.319	2.871	24.3	59.13	9,618.37	584.082	91.134	18.693	121.086
S 20	199.585	0.9	30.672	82.296	14,524.31	819.99	98.568	33.309	146.772
S 21	203.693	0.639	16.65	43.056	7,294.59	485.379	54.909	29.538	96.039
S 22	196.094	1.089	29.619	74.349	12,340.02	744.291	94.824	35.613	144.594
S 23	221.64	0.54	10.458	18.909	4,289.21	591.318	36.306	30.969	59.418
S 24	196.807	0.828	17.037	25.938	6,732.00	778.158	51.417	31.014	87.309
S 25	272.574	0.855	12.24	29.259	7,760.67	1002.942	37.989	33.327	124.938
S 26	225.178	1.026	13.842	19.971	4,750.87	849.078	41.022	32.472	87.237
Minimum	183.39	0.54	10.46	18.91	4289	424.20	36.31	16.09	59.42
Maximum	272.57	16.02	33.46	217.78	14623	1002.94	201.47	58.10	1201.77
Mean	**210.07**	**6.50**	**23.97**	**87.15**	**10706**	**697.53**	**112.43**	**31.11**	**207.40**
Standard deviation	20.23	5.62	5.96	47.69	2855	138.46	43.68	8.66	216.76
Upper continental crust [83]	1.5	0.098	10	25	35000	600	20	20	71
Mean concentration of heavy metals in rocks [84]	5.5 - 12	0.01 - 2.6	1.3 - 10	9.9-39	14000 - 28000	990 - 7400	1.8 - 18	2.6 - 27	37- 68
[82]	5.80	7.50	300	16	–	740	91	20	240

In this research, the mean concentration of As was 210.07 ± 20.32 mg kg^-1^, which was greater than in previous studies (Table 6). When compared to other research, the mean Cd concentration was greater, but lower than the reported value of 11.26 mg kg^-1^ in soils by Shokr et al., [44].The current study’s Co content was higher than the mean values in prior investigations, as shown in Table 6. The current study found a higher mean Cr value compared to earlier studies, although lower than Abuzaid [85] et al.’s concentration in Egypt’s North Nile Delta. Fe concentration in the current study is lower than that in soil samples from other study regions (Table 6). Mn concentrations in soils in Al-Baha area, Saudi Arabia, were greater than the current study (Table 6). The levels of Ni reported by Kelepertzis [86]for agricultural soils in the Argolida basin, Greece (mean: 146.80 mg/kg) are greater than those found in this study. The current study found a mean Pb concentration of 31.11 mg kg^-1^ in topsoils of the study area, which was higher than the mean values reported in literature (Table 6). However, the values were lower than those reported in soils by Shokr et al., [44], Abuzaid et al., [85], and Mohammed et al., [87]. Table 6 shows that the average Zn contents in other investigations were lower than those found in soil samples of the current investigation.

**Table 6:** Comparison of the current study’s HM concentration with that of other soils worldwide.

	As	Cd	Co	Cu	Fe	Mn	Ni	Pb	Zn	References
Kafr El-Sheikh Governorate, Egypt	183.39-272.57	0.54-16.02	10.46-33.46	18.91-217.78	4289-14623	424.20-1002.94	36.31-201.47	16.09-58.10	59.42-1201.77	Current study
Almería, Spain	–	0.4	–	25.7	–	–	26.90	25.60	65.70	[88]
Argolida basin, Greece	–	0.5	–	74.70	–	–	146.80	19.70	74.90	[86]
Beijing, China	–	0.1	–	27.70	–	–	24.60	30	77.80	[89]
Middle Nile delta, Egypt	–	11.26	–	–	–	–	–	54.15	61.29	[44]
North Nile delta, Egypt	–	2.27	5.93	311.22	–	–	46.74	104.21	149.27	[85]
South Al-shrkia Governorate, Egypt	208.78	1.48	–	–	19565	–	45.39	40.80	191.56	[87]
Al-Lith, Saudi Arabia	1.67		10.12	21.69			18.29	2.69	43.69	[90]
Al-Baha region, Saudi Arabia	7.10	0.80	20.60	61.10	45846	1015.20	42.30	10.10	96.70	[91]
Jazan, Saudi Arabia	2.94	–	12.31	24.11	32508	–	30.17	4.97	50.40	[33]

### 3.3. Statistical analysis of studied HMs

#### 3.3.1. Correlation between HMs in the soil samples.

**Fig 7:**
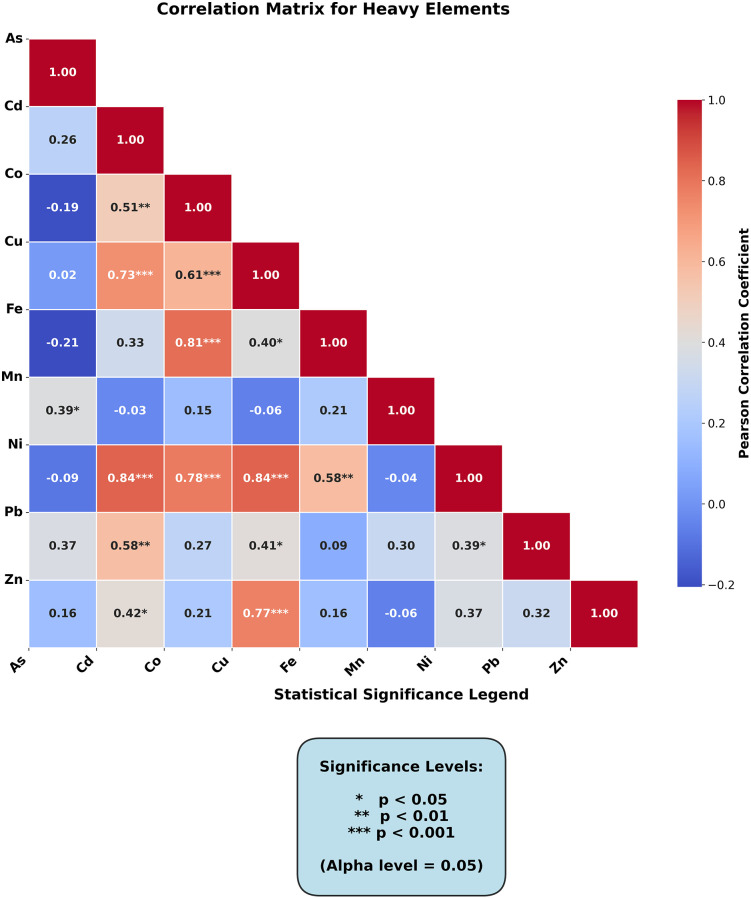
Pearson’s correlation matrix for the dataset.

Pearson’s correlation matrix for all soil sample counts (n = 27) shows significant (p < 0.05) and extremely significant (p < 0.01) associations between heavy metals. The metals analyzed showed positive connections with one another, indicating that they came from the same source [92] (Fig 7).

#### 3.3.2. Potential sources of the HMs.

One popular statistical technique for determining potential sources of heavy metals (oid) in soil is principal component analysis (PCA) [71,93,94]. When the KMO value was more than 0.5 and p < 0.001 in the Bartlett sphericity tests, PCA was performed. Three principal components with an 80.36% total variance were revealed by the PCA analysis and have eigenvalues greater than 1 (Fig 7). The PC1 included primarily heavy loading of metal (oid)s and explained 45.76% of the overall variation. In contrast, PC2 included positive loading of As and Pb and explained 19.59% of the total variation. Anthropogenic activities may have contributed to the increased Pb content [95]. The amount and distribution of Pb in soils may be primarily caused by fertilizers and vehicle emissions [96]. Arsenic in soil is typically thought to be primarily geological or organic, with clayey soils having a greater background concentration [97]. Nonetheless, because arsenic released from man-made sources greatly outweighs that from natural sources, anthropogenic arsenic pollution is rather common [79]. The third is that PC has Mn positively loaded, the majority of this metal came from parent material in agricultural soil, according to Cheng et al., [98] (Fig 7) A relatively greater amount of Mn and Zn, along with pollution indices, points to the potential for human interference in agricultural soil. Possible sources of metal(oid)s include transportation activities like spraying, ploughing, and harvesting, as well as agricultural activities like chemical fertilizers, insecticides, or herbicides and atmospheric deposition. Previous research has shown a correlation between agricultural activities and atmospheric deposition and the concentrations of Cd, Cu, and Zn in agricultural soils [71,99–102].

**Fig 8:**
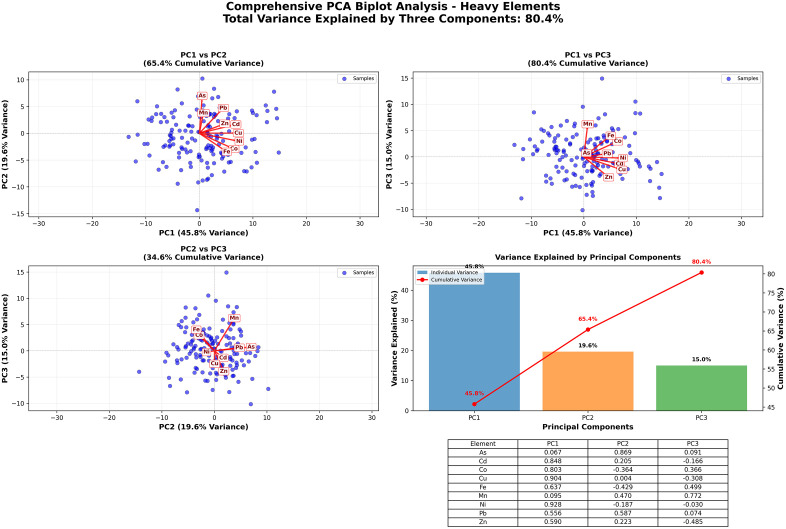
PCA biplot of the examined area.

#### 3.3.3. Cluster analysis.

**Fig 9:**
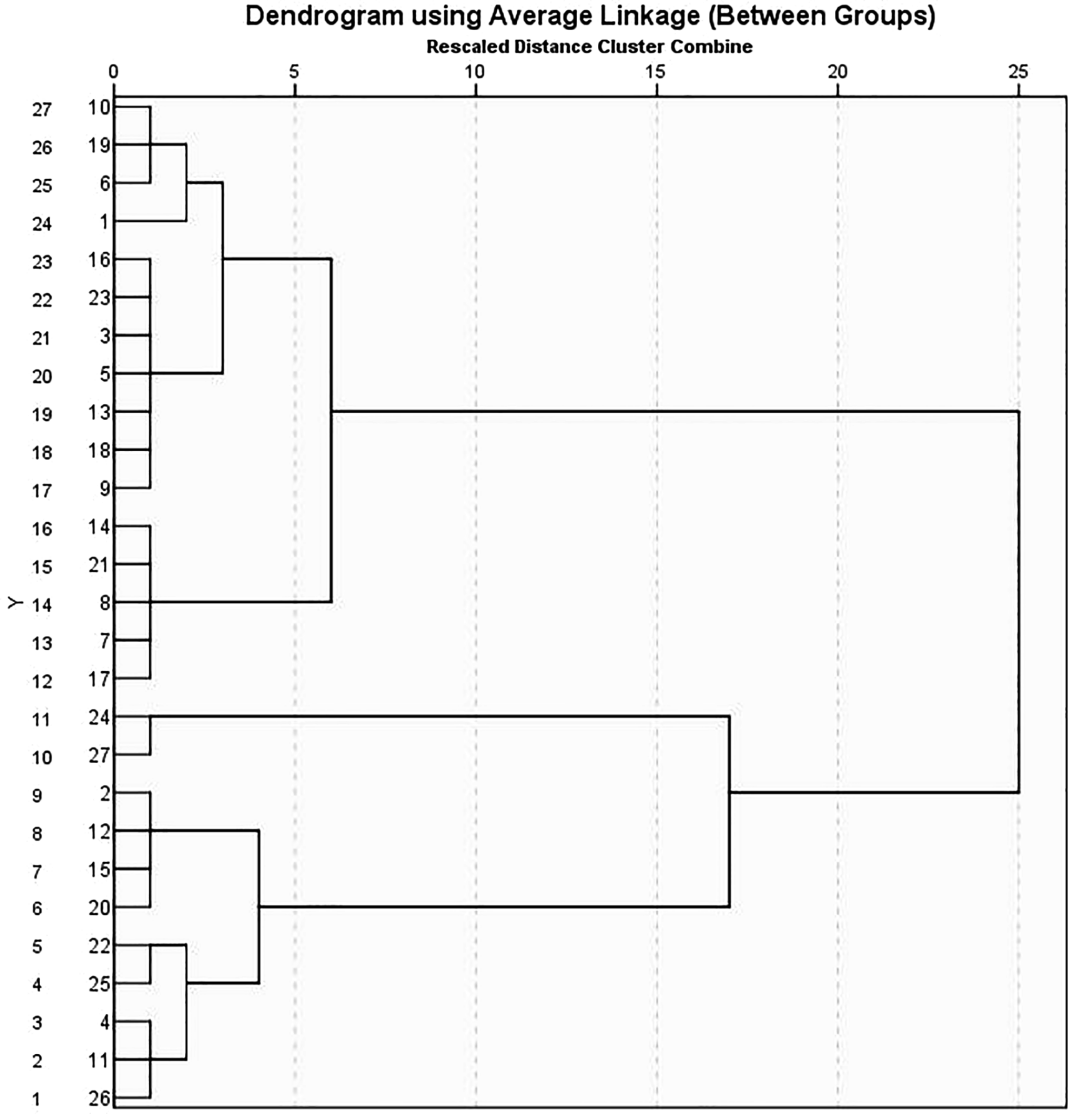
Dendrogram of studied HMs.

Based on the amounts of heavy metals, the cluster analysis results clearly separated the soil samples into two main groups (C1 and C2) (Fig 9). The mean values of numerous elements were greater in Cluster 1 (C1), especially Fe (12,655.46 mg kg^-1^), Mn (725.76 mg kg^-1^), Zn (246.23 mg kg^-1^), and As (209.66 mg kg^-1^)Table 7. This suggests that the soils in this cluster have higher levels of major and trace element enrichment, which could be due to either potential anthropogenic contributions like industrial processes, wastewater irrigation, or agricultural inputs. This cluster’s high concentrations of Pb (32.45 mg kg^-1^) and Cd (8.01 mg kg^-1^) further indicate possible pollution hotspots that could provide health and environmental risks. The cluster 1 samples are located near the textile mills at El-Mehalla El-Kobra. Therefore, anthropogenic outputs from household items and industrial effluents—especially those from paint, sewage, leather tanning, and agricultural wastes—especially those containing superphosphate fertilizers and pesticides—may be the source of pollution in this area [34]. However, the majority of elements, such as Fe (7,870.65 mg kg^-1^), Zn (150.93 mg kg^-1^), Pb (29.17 mg kg^-1^), and Cd (4.31 mg kg^-1^), are found in relatively lower amounts in Cluster 2 (C2). Despite the fact that the two clusters’ As values are comparable, the group’s generally lower C2 concentrations imply that the soils in this group are less contaminated (Table 7). These results are corroborated by the standard deviation values, which indicate significant variability in Mn (167.72), which may be related to their diverse distribution across various soil conditions and their close relationship with soil mineralogy. Lower variability was seen in elements like Cd and Pb, indicating more reliable but confined input sources. These results align with previous research [103–105], which verified that the Nile Delta soils contain Mn from a similar source. Mn is naturally present in ferromagnesian minerals such as olivine, hornblende, and augite, because they can easily replace magnesium (II) due to their different divalent radii. As a result, basic (basalt) and ultrabasic (serpentine) rocks contain the highest concentration of Mn [106]. Thus, a significant source of this metal in the Nile Delta area is the Nile sediments, which are abundant in serpentine and basalt rocks [107].

**Table 7:** Cluster analysis of studied HMs within study area.

Clusters	As	Cd	Co	Cu	Fe	Mn	Ni	Pb	Zn
C1	209.66 ± 17.80	8.01 ± 5.68	26.88 ± 4.07	98.32 ± 38.63	12655.46 ± 1269.99	725.76 ± 111.31	126.88 ± 29.34	32.45 ± 9.31	246.23 ± 256
C2	210.67 ± −24.26	4.31 ± 5.00	19.74 ± 5.86	70.90 ± 56.44	7870.65 ± 1963.12	656.48 ± 167.72	91.41 ± 53.38	29.17 ± 7.59	150.93 ± 104.41

### 3.5. Degree of contamination within study area

The research area’s soil had varying levels of contamination, ranging from uncontaminated to highly contaminated. According to I_geo_, all soil tests containing As were classified as extremely polluted. This indicated that the soils contained much higher quantities of As compared to the background. The I_geo_ of Cd ranged from 1.88 to 6.77, with a mean value of 4.79, indicating strongly to extremely contaminated (Table 8). The lowest values of Igeo for Co, Cu, Fe, Mn, Ni, Pb, and Zn were −0.52, −0.99, −1.36, −1.09, 0.28, −0.90, and −0.84, respectively, with the highest values being 1.16, 2.54, 2.48, 0.16, 2.75, and 0.95 (Table 8). Suggested that the soils of the study area fell into uncontaminated and strongly to extremely contaminated classes.

**Table 8:** Contamination indices statistics of the study area.

EF									
	As	Cd	Co	Cu	Fe	Mn	Ni	Pb	Zn
Min	17.07	5.41	0.32	0.38	–	0.13	0.13	0.78	0.13
Max	253.39	25.34	2.32	2.24		2.01	2.01	3.57	2.66
Mean	58.04	12.42	0.82	0.97	–	0.48	0.48	1.62	0.64
STD	68.74	6.99	0.72	0.63	–	0.56	0.56	1.04	0.73
	CF								
Min	122.26	5.51	1.05	0.76	0.58	0.71	1.82	0.8	0.84
Max	181.72	163.47	3.35	8.71	8.36	1.67	10.07	2.9	16.93
Mean	140.05	66.37	2.4	3.49	4.79	1.16	5.62	1.56	2.92
STD	13.49	57.4	0.6	1.91	2.67	0.23	2.18	0.43	3.05
I_geo_									
Min	6.35	1.88	−0.52	−0.99	−1.36	−1.09	0.28	−0.9	−0.84
Max	6.92	6.77	1.16	2.54	2.48	0.16	2.75	0.95	3.5
Mean	6.53	4.79	0.65	1.06	1.35	−0.41	1.82	−0.01	0.65
STD	1.26	1.86	0.42	0.83	1.26	0.29	0.72	0.4	0.87
ER									
Min	–	165.31	5.23	3.78	–	0.71	9.08	4.02	0.84
Max	–	4904.08	16.73	43.56	–	1.67	50.37	14.52	16.93
Mean	–	1991.02	11.98	17.43	–	1.16	28.11	7.78	2.92
STD		1750.55	3.21	10.55		0.25	11.68	2.50	3.89

The EF of As fluctuated between 17.07 and 253.39, with an average value of 58.04, indicating severe enrichment. The EF of Cd ranges from 5.41 to 25.34, placing it in the considerable and very high enrichment category (Table 8). Co and Cu have EFs ranging from 0.32 to 2.32 and 0.38 to 2.24, respectively, placing them in the natural and moderate groups. The lowest values of EF for Mn, Ni, Pb, and Zn were 0.13, 0.78, 0.13, and 0.19, while the highest values were 2.01, 3.57, 2.66, and 2.55, respectively, placing them in the natural and moderate categories. The values of CF in soil were reported as follows: As ranged from 122.26 to 181.72 with an average value 140, Cd ranged from 5.51 to 163.47 with an average 66.37, Co ranged from 1.05 to 3.35 with an average value 2.40, Cu ranged from 0.76 to 8.71 with an average 3.49, Fe ranged from 0.58 to 8.36 with an average 4.79, Mn ranged from 0.71 to 1.67 with an average 1.16, Ni ranged from 1.82 to 10.07 with an average 5.62, pb ranged from 0.80 to 2.90 with an average 1.16,and Zn ranged from 0.84 to 16.93 with an average 2.92. The CF mean results revealed that the soils in the research area were in moderate to extremely high pollution status (Table 8).

**Fig 10:**
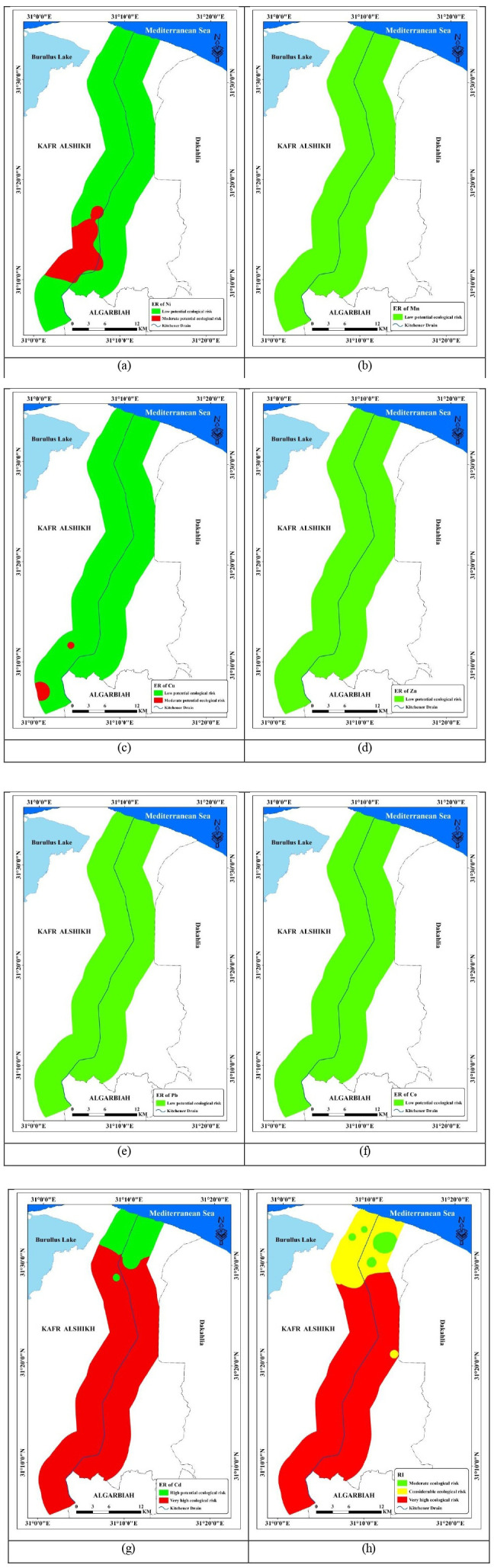
Spatial distribution of ER and RI in the investigated area.

Using the greatest value of the heavy metal Tr reported in the literature, the ecological risk of individual heavy metals (ER) has been calculated for soil samples [54–57], (Fig 9) displays the computed Er and RI values. Cd > Ni > Cu > Co > Pb > Zn > Mn was the decreasing order of the Er level, which was determined by the median levels of Ni, Mn, Cu, Zn, Pb, Cd, and Co, which were 28.10, 1.16, 17.42, 2.92, 7.77, 1991, and 11.98, respectively. All metals (oid) have minimal ecological danger according to the ER results, with the exception of Cd, which had high and very high ecological risk, and Cu, and Ni, which in some locations had moderate ecological risk. Er of metal (oid)s were added up to determine the RI values. With a median value of 2060.40 and a range of 192.95 to 5006.97, the RI values showed a moderate, considerable, and very high ecological risk (Fig 10).

### 3.6. Health risk assessment

#### 3.6.1. Non-carcinogenic risk.

The study estimated the non-carcinogenic risk of five metalloids found in soil samples for children and adults by ingestion, inhalation, and skin exposure. The mean levels of each element supplied in Table 9 have been substituted into formulas. The HQ and HI values related to metal(loid) exposure for children and adults from soil are given in Table 9. The mean HI of As was 9.014 for children and 0.969 for adults. The mean HI of Cd was 0.106 for children and 0.012 for adults. The mean HI of Co was 0.015 for children and 0.002 for adults. The average HI values for Cu, Fe, Mn, Ni, Pb, and Zn were 0.028, 0.198, 0.065, 0.072, 0.116, 0.009, and 0.003 for children and adults, respectively. Through three channels, the HQ values of various metals decreased in the following order: ingestion, dermal absorption, and inhalation. This finding suggests that the main way that HMs that are harmful to human health enter the body is through the ingestion of soil particles. Similar findings have been found in earlier research [108,109].

**Table 9:** Non-cancer health risk indices due to exposure to heavy metals in the soil samples.

Risk	Pathway		As	Cd	Co	Cu	Fe	Mn	Ni	Pb	Zn
HQ (children)	Ingestion	Min.	7.815	0.007	0.007	0.006	0.078	0.039	0.023	0.059	0.003
		Max.	11.617	0.205	0.021	0.070	0.267	0.092	0.129	0.212	0.051
		Mean	8.953	0.083	0.015	0.028	0.196	0.064	0.072	0.114	0.009
	Inhalation	Min.	2.18E-10	1.93E-13	6.54E-10	1.68E-13	1.92E-12	1.06E-08	6.3E-13	1.63E-12	7.08E-14
		Max.	3.25E-10	5.72E-12	2.09E-09	1.94E-12	6.53E-12	2.51E-08	3.49E-12	5.9E-12	1.43E-12
		Mean	2.5E-10	2.32E-12	1.5E-09	7.74E-13	4.78E-12	1.74E-08	1.95E-12	3.16E-12	2.47E-13
	Dermal contact	Min.	5.3E-02	1.9E-03	2.3E-05	5.6E-05	1.1E-03	6.5E-04	1.5E-06	1.1E-03	3.5E-05
		Max.	7.9E-02	5.7E-02	7.5E-05	6.5E-04	3.7E-03	1.5E-03	8.6E-06	4.0E-03	7.2E-04
		Mean	6.1E-02	2.3E-02	5.4E-05	2.6E-04	2.7E-03	1.1E-03	4.8E-06	2.1E-03	1.2E-04
HI		Min.	7.869	0.009	0.007	0.006	0.079	0.039	0.023	0.060	0.003
		Max.	11.696	0.262	0.021	0.070	0.271	0.093	0.129	0.216	0.052
		Mean	9.014	0.106	0.015	0.028	0.198	0.065	0.072	0.116	0.009
HQ (adult)	Ingestion	Min.	0.837	0.001	0.001	0.001	0.008	0.004	0.002	0.006	0.000
		Max.	1.245	0.022	0.002	0.007	0.029	0.010	0.014	0.023	0.005
		Mean	0.959	0.009	0.002	0.003	0.021	0.007	0.008	0.012	0.001
	Inhalation	Min.	1.2E-10	1.2E-10	3.7E-10	9.5E-14	1.1E-12	6.0E-09	1.7E-06	9.2E-13	4.0E-14
		Max.	1.8E-10	1.8E-10	1.2E-09	1.1E-12	3.7E-12	1.4E-08	9.7E-06	3.3E-12	8.1E-13
		Mean	1.4E-10	1.4E-10	8.5E-10	4.4E-13	2.7E-12	9.8E-09	5.4E-06	1.8E-12	1.4E-13
	Dermal contact	Min.	8.1E-03	3.0E-04	3.6E-06	8.6E-06	1.7E-04	1.0E-04	3.7E-05	1.7E-04	5.4E-06
		Max.	1.2E-02	8.8E-03	1.1E-05	9.9E-05	5.7E-04	2.4E-04	2.0E-04	6.0E-04	1.1E-04
		Mean	9.3E-03	3.6E-03	8.2E-06	4.0E-05	4.2E-04	1.6E-04	1.1E-04	3.2E-04	1.9E-05
HI		Min.	0.846	0.001	0.001	0.001	0.009	0.004	0.003	0.006	0.000
		Max.	1.257	0.031	0.002	0.008	0.029	0.010	0.014	0.023	0.006
		Mean	0.969	0.012	0.002	0.003	0.021	0.007	0.008	0.013	0.001

Children and adults had HQ and HI values lower than one in all three exposure pathways, with the exception of As in the Ingestion pathway for children. The study found no acute non-cancer harm from soil pollution, except for As in the investigated population.

#### 3.6.2. Cancer risks (CR).

**Fig 11:**
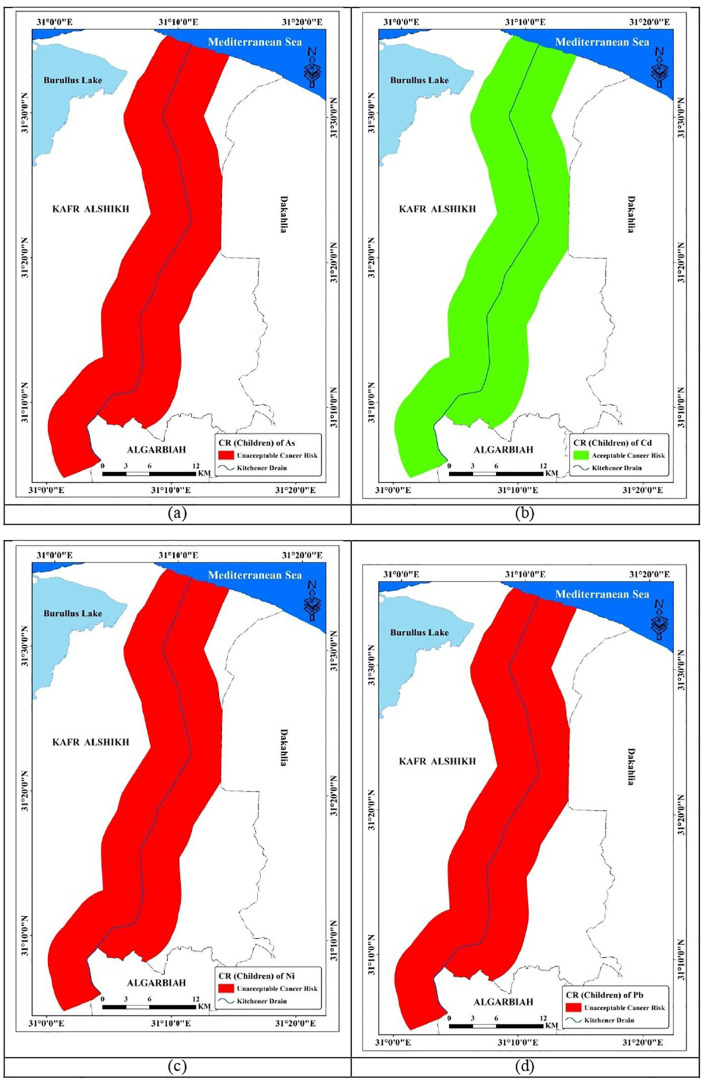
Spatial distribution of CR total for children within study area.

**Fig 12:**
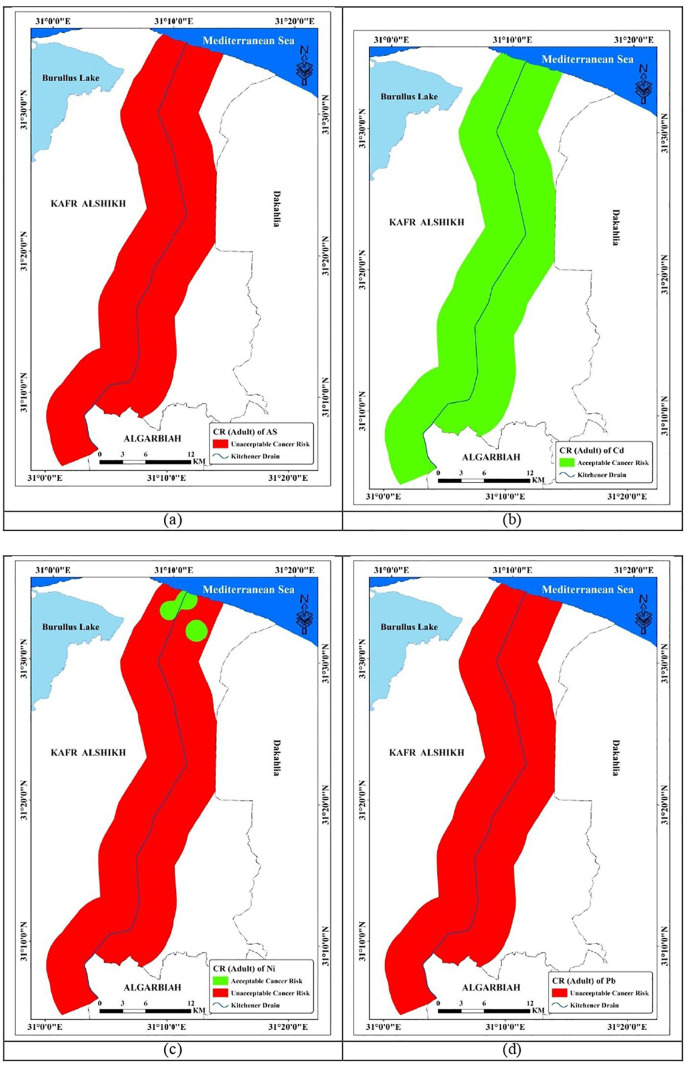
Spatial distribution of CR total for children within study area.

Many scientists worldwide are concerned about the transmission of metals from soil to human bodies [110–112]. This study assessed the cancer risk of elements such as As, Cd, Ni, and Pb. Table 10 summarizes the cancer risks associated with metal(loid) exposure in soil. The mean CR total (sum of ingestion, inhalation, and dermal contact pathways for each element) related to children’s exposure to the examined elements in soil samples of the study area was in the following order: Pb (4.5E-02)>>As (4.1E-03)> Ni (2.6E-03)> Cd (4.7E-05). As a result, Pb in soil was assessed to pose the highest cancer risk. The research area’s soil contains As, Pb, and Ni which pose a high cancer risk (CR total > 1.00E-04) [69,70]. The average CR total associated with adult exposure to the studied elements in soil samples from the study area was in the following order: Pb (4.8E-03)> As (4.4E-04), Ni (2.9E-04), and, Cd (5.3E-06).Pb is implicated, including encephalopathy, kidney illness, and neuropathy, as shown in Figs 11 and 12 [113]

**Table 10:** Cancer health risk indices statistics.

Risk	pathway		As	Cd	Ni	Pb
CR (children)	Ingestion	Min.	3.5E-03	3.5E-06	7.9E-04	3.7E-06
		Max.	5.2E-03	1.0E-04	4.4E-03	8.7E-02
		Mean	4.0E-03	4.2E-05	2.4E-03	4.5E-02
	Inhalation	Min.	9.9E-13	1.2E-15	1.1E-14	2.4E-16
		Max.	1.5E-12	3.6E-14	6.0E-14	8.7E-16
		Mean	1.1E-12	1.5E-14	3.4E-14	4.7E-16
	Dermal contact	Min.	2.4E-05	3.9E-07	5.5E-05	1.0E-08
		Max.	3.6E-05	1.1E-05	3.1E-04	2.4E-04
		Mean	2.8E-05	4.7E-06	1.7E-04	1.3E-04
CR Total		Min.	3.5E-03	3.9E-06	8.4E-04	3.7E-06
		Max.	5.3E-03	1.2E-04	4.7E-03	8.8E-02
		Mean	**4.1E-03**	**4.7E-05**	**2.6E-03**	**4.5E-02**
CR (adult)	Ingestion	Min.	3.8E-04	3.8E-07	8.5E-05	4.0E-07
		Max.	5.6E-04	1.1E-05	4.7E-04	9.4E-03
		Mean	4.3E-04	4.5E-06	2.6E-04	4.8E-03
	Inhalation	Min.	5.6E-13	6.9E-16	6.1E-15	1.4E-16
		Max.	8.3E-13	2.0E-14	3.4E-14	4.9E-16
		Mean	6.4E-13	8.3E-15	1.9E-14	2.6E-16
	Dermal contact	Min.	3.7E-06	5.9E-08	8.4E-06	1.6E-09
		Max.	5.5E-06	1.8E-06	4.7E-05	3.7E-05
		Mean	4.2E-06	7.1E-07	2.6E-05	1.9E-05
CR total		Min.	3.8E-04	4.4E-07	9.3E-05	4.0E-07
		Max.	5.7E-04	1.3E-05	5.2E-04	9.4E-03
		Mean	4.4E-04	5.3E-06	2.9E-04	4.8E-03

### 3.7. Limitations of the study

Despite offering insightful information, this study has a number of drawbacks. First, the availability and spatial resolution of input data from monitoring stations limit the pollution index’s accuracy. Second, as personal exposure was calculated using residence location rather than real mobility patterns, the exposure assessment is probably affected by the Uncertain Geographic Context Problem. Lastly, although required, the interpolation techniques employed in GIS add a measure of uncertainty to the estimation of pollution levels throughout the research region.

## 4. Conclusion

Metal(loid) pollution in soil is a global environmental concern, posing risks to agriculture and human health. This study indicated that Fe had the highest metal(loid) contamination in the Nile delta soil, followed by Mn, As, Zn, Ni, Cu, Pb, Co, and Cd levels. The CF, Igeo, and EF indices all show that the soils in the studied region were contaminated with metals. Pesticides and chemical fertilizers are potential sources of metals in soil, with the exception of Mn, which may come from a natural source.

Children and adults had HQ and HI values less than one (threshold) in all three exposure paths, with the exception of As in the ingestion pathway for children. Except for As, the study identified no acute non-cancer damage from soil contamination in the population studied. Furthermore, the soil at the research site contains As, and Pb, all of which have a high cancer risk (CR > 1.00E-4).The research’s findings can serve as a standard for authorities to assess metal(loid) contamination, human health hazards, and controlling factors for metal(loid) buildup in Nile delta soil. This approach improves soil management, removal, and deterioration control by providing current and comprehensive results. As a result, further research into metal(loid)s in the area’s soil is strongly advised to ensure food safety. This study supports the Sustainable Development Goals (SDGs) of the UN, especially those that deal with sustainable land management and environmental preservation. The research helps achieve SDG 2 (Zero Hunger) by promoting sustainable agricultural productivity; SDG 3 (Good Health and Well-Being) by lowering health risks related to soil pollutants; SDG 6 (Clean Water and Sanitation) by preventing the transfer of contaminants to surface and groundwater; and SDG 15 (Life on Land) by preventing land degradation and desertification. This is achieved by evaluating soil contamination and its spatial distribution. Therefore, in accordance with the global 2030 Agenda, the study’s findings offer insightful information for environmentally friendly soil usage and conservation. More soil samples and sophisticated geostatistical models (such as co-kriging and machine learning-based spatial prediction) can be used in future studies to build on this one and map contaminants more precisely. Long-term monitoring initiatives should also be created to evaluate changes in pollutant dynamics and soil quality over time. Collaborative research relating soil contamination to climate change scenarios and land management techniques would also advance the science and aid in policymaking.

Certain management strategies to lessen the adverse effects of soil pollution, as demonstrated by the items that follow.

1- Switching from growing crops with greater accumulation rates, like leafy vegetables like spinach and lettuce, to less sensitive ones, like pulses, tubers, and specific grains, risk is decreased.
2- The rate of absorption can also be affected by crop rotation. The bioavailability of a trace element during the subsequent cropping cycle may be impacted by the rhizosphere impacts of some plants.
3- Microorganisms and plants can be used in biological procedures, because they are more economical and have the potential to eliminate pollutants without producing secondary toxins,
Increasing community knowledge of the site’s hazards and the usage of pesticides.
4- Restoring a nearby medical center to provide health care services to the population that experienced adverse health effects

## Supporting information

S1 File Supplementary data. (DOCX)

## References

[1] Sezgin N, Kinda S, Temelli UE, Sezgin N. Pollution indices assessment of metal concentrations in Karabuk soil samples. International Journal of Agriculture Environment and Food Sciences. 2023;7(2):384–98. doi: 10.31015/jaefs.2023.2.17

[2] Zemmouri B, Lammoglia S-K, Bouras F-Z, Seghouani M, Rebouh NY, Latati M. Modelling human health risks from pesticide use in innovative legume-cereal intercropping systems in Mediterranean conditions. Ecotoxicol Environ Saf. 2022;238:113590. doi: 10.1016/j.ecoenv.2022.113590 35525117

[3] Mishra RK, Mohammad N, Roychoudhury NJVS. Soil pollution: Causes, effects and control. 2016;3(1):1–14.

[4] Khan S, Naushad M, Lima EC, Zhang S, Shaheen SM, Rinklebe JJ. Global soil pollution by toxic elements: Current status and future perspectives on the risk assessment and remediation strategies–A review. J Hazard Mater. 2021;417:126039. 34015708 10.1016/j.jhazmat.2021.126039

[5] Sajjadi SA, Mohammadi A, Khosravi R, Zarei A. Distribution, exposure, and human health risk analysis of heavy metals in drinking groundwater of Ghayen County, Iran. Geocarto International. 2022;37(26):13127–44. doi: 10.1080/10106049.2022.2076916

[6] Vidal O, Rostom F, François C, Giraud G. Global Trends in Metal Consumption and Supply: The Raw Material–Energy Nexus. Elements. 2017;13(5):319–24. doi: 10.2138/gselements.13.5.319

[7] Mohamed NNJTND. Land degradation in the Nile Delta; 2016.

[8] Rashid A, Khattak SA, Ali L, Zaib M, Jehan S, Ayub M, et al. Geochemical profile and source identification of surface and groundwater pollution of District Chitral, Northern Pakistan. Microchemical Journal. 2019;145:1058–65. doi: 10.1016/j.microc.2018.12.025

[9] Hashim T, Abbas HH, Farid IM, El-Husseiny O, Abbas MH. Accumulation of some heavy metals in plants and soils adjacent to Cairo–Alexandria agricultural highway. E J Soil Sci. 2017;57(2):215–32.

[10] Abou El-Anwar EAJ. Assessment of heavy metal pollution in soil and bottom sediment of Upper Egypt: comparison study. NRC. 2019;43:1–11.

[11] Abou El-Anwar E, Samy Y, Salman S. Heavy metals hazard in Rosetta Branch sediments, Egypt. SJJMES. 2018;9(7):2142–52.

[12] Khalifa M, Gad A. Assessment of Heavy Metals Contamination in Agricultural Soil of Southwestern Nile Delta, Egypt. Soil and Sediment Contamination: An International Journal. 2018;27(7):619–42. doi: 10.1080/15320383.2018.1498445

[13] Aitta A, El-Ramady H, Alshaal T, El-Henawy A, Shams M, Talha N, et al. Seasonal and Spatial Distribution of Soil Trace Elements around Kitchener Drain in the Northern Nile Delta, Egypt. Agriculture. 2019;9(7):152. doi: 10.3390/agriculture9070152

[14] Abu Khatita AM, Koch R, Bamousa AO. Sources identification and contamination assessment of heavy metals in soil of Middle Nile Delta, Egypt. Journal of Taibah University for Science. 2020;14(1):750–61. doi: 10.1080/16583655.2020.1771833

[15] Huang S, Shao G, Wang L, Wang L, Tang L. Distribution and Health Risk Assessment of Trace Metals in Soils in the Golden Triangle of Southern Fujian Province, China. Int J Environ Res Public Health. 2018;16(1):97. doi: 10.3390/ijerph16010097 30602676 PMC6339116

[16] Zhang R, Chen T, Zhang Y, Hou Y, Chang Q. Health risk assessment of heavy metals in agricultural soils and identification of main influencing factors in a typical industrial park in northwest China. Chemosphere. 2020;252:126591. doi: 10.1016/j.chemosphere.2020.126591 32240858

[17] Doležalová Weissmannová H, Mihočová S, Chovanec P, Pavlovský J. Potential Ecological Risk and Human Health Risk Assessment of Heavy Metal Pollution in Industrial Affected Soils by Coal Mining and Metallurgy in Ostrava, Czech Republic. Int J Environ Res Public Health. 2019;16(22):4495. doi: 10.3390/ijerph16224495 31739633 PMC6888271

[18] Chen H, Wang L, Hu B, Xu J, Liu X. Potential driving forces and probabilistic health risks of heavy metal accumulation in the soils from an e-waste area, southeast China. Chemosphere. 2022;289:133182. doi: 10.1016/j.chemosphere.2021.133182 34883131

[19] Obiri-Nyarko F, Duah AA, Karikari AY, Agyekum WA, Manu E, Tagoe R. Assessment of heavy metal contamination in soils at the Kpone landfill site, Ghana: Implication for ecological and health risk assessment. Chemosphere. 2021;282:131007. doi: 10.1016/j.chemosphere.2021.131007 34087555

[20] Doabi SA, Karami M, Afyuni M, Yeganeh M. Pollution and health risk assessment of heavy metals in agricultural soil, atmospheric dust and major food crops in Kermanshah province, Iran. Ecotoxicol Environ Saf. 2018;163:153–64. doi: 10.1016/j.ecoenv.2018.07.057 30053585

[21] Zhang Y, Wang S, Gao Z, Zhang H, Zhu Z, Jiang B, et al. Contamination characteristics, source analysis and health risk assessment of heavy metals in the soil in Shi River Basin in China based on high density sampling. Ecotoxicol Environ Saf. 2021;227:112926. doi: 10.1016/j.ecoenv.2021.112926 34687942

[22] Pecina V, Brtnický M, Baltazár T, Juřička D, Kynický J, Vašinová Galiová M. Human health and ecological risk assessment of trace elements in urban soils of 101 cities in China: A meta-analysis. Chemosphere. 2021;267:129215. doi: 10.1016/j.chemosphere.2020.129215 33359981

[23] Wu W, Wu P, Yang F, Sun D-L, Zhang D-X, Zhou Y-K. Assessment of heavy metal pollution and human health risks in urban soils around an electronics manufacturing facility. Sci Total Environ. 2018;630:53–61. doi: 10.1016/j.scitotenv.2018.02.183 29475113

[24] Bai J, Zhao X. Ecological and Human Health Risks of Heavy Metals in Shooting Range Soils: A Meta Assessment from China. Toxics. 2020;8(2):32. doi: 10.3390/toxics8020032 32370002 PMC7356891

[25] Saraswat A, Ram S, Raza MB, Islam S, Sharma S, Omeka ME, et al. Potentially toxic metals contamination, health risk, and source apportionment in the agricultural soils around industrial areas, Firozabad, Uttar Pradesh, India: a multivariate statistical approach. Environ Monit Assess. 2023;195(7):863. doi: 10.1007/s10661-023-11476-3 37336819

[26] Li Y, Zhu Q, Tang X, Wang C, Zhai S. Ecological and Health Risk Assessment of Heavy Metals in Farmland in the South of Zhangbei County, Hebei Province, China. Applied Sciences. 2022;12(23):12425. doi: 10.3390/app122312425

[27] Gui H, Yang Q, Lu X, Wang H, Gu Q, Martín JD. Spatial distribution, contamination characteristics and ecological-health risk assessment of toxic heavy metals in soils near a smelting area. Environ Res. 2023;222:115328. doi: 10.1016/j.envres.2023.115328 36693463

[28] Shokr MS, El Baroudy AA, Fullen MA, El-beshbeshy TR, Ramadan AR, Abd El Halim A, et al. Spatial distribution of heavy metals in the middle nile delta of Egypt. International Soil and Water Conservation Research. 2016;4(4):293–303. doi: 10.1016/j.iswcr.2016.10.003

[29] Omran E. Environmental modelling of heavy metals using pollution indices and multivariate techniques in the soils of Bahr El Baqar, Egypt. E-SEJMES. 2016;2:1–17.

[30] El-Zeiny AM, Abd El-Hamid HT. Environmental and human risk assessment of heavy metals at northern Nile Delta region using geostatistical analyses. The Egyptian Journal of Remote Sensing and Space Science. 2022;25(1):21–35. doi: 10.1016/j.ejrs.2021.12.005

[31] Mohamed ES, Belal A, Shalaby A. Impacts of soil sealing on potential agriculture in Egypt using remote sensing and GIS techniques. Eurasian Soil Sc. 2015;48(10):1159–69. doi: 10.1134/s1064229315100075

[32] Abuzaid AS, El-Husseiny AMJ. Modeling crop suitability under micro irrigation using a hybrid AHP-GIS approach. Agricultural Journal. 2022;15(13):1217.

[33] Kahal AY, El-Sorogy AS, Meroño de Larriva JE, Shokr MS. Mapping Soil Contamination in Arid Regions: A GIS and Multivariate Analysis Approach. Minerals. 2025;15(2):124. doi: 10.3390/min15020124

[34] Hendawy E, Belal AA, Sheta AEAS, Mohamed ES, Kucher DE, Jalhoum ME. Assessment of human activities on soil contamination in Egypt: implications for the MENA region. 2025;13:1493197.

[35] Hammam AA, Mohamed ES. Mapping soil salinity in the East Nile Delta using several methodological approaches of salinity assessment. The Egyptian Journal of Remote Sensing and Space Science. 2020;23(2):125–31. doi: 10.1016/j.ejrs.2018.11.002

[36] Abuzaid AS, Bassouny MAJET. Total and DTPA-extractable forms of potentially toxic metals in soils of rice fields, north Nile Delta of Egypt. 2020;18:100717.

[37] Abowaly ME, Belal A-AA, Abd Elkhalek EEA, Elsayed S, Abou Samra RMA, Alshammari AS, et al. Assessment of Soil Pollution Levels in North Nile Delta, by Integrating Contamination Indices, GIS, and Multivariate Modeling. Sustainability. 2021;13(14):8027. doi: 10.3390/su13148027

[38] Department A. Keys to soil taxonomy. Government Printing Office; 2014.

[39] Baroudy AAE, Ali AM, Mohamed ES, Moghanm FS, Shokr MS, Savin I, et al. Modeling Land Suitability for Rice Crop Using Remote Sensing and Soil Quality Indicators: The Case Study of the Nile Delta. Sustainability. 2020;12(22):9653. doi: 10.3390/su12229653

[40] El Shazly EM. The geology of the Egyptian region. The ocean basins and margins: Volume 4A The Eastern Mediterranean. Springer. 1977. p. 379–444.

[41] Hemdan GJAE-K. Personality of Egypt, a study in uniqueness of the position. 1980;1:841.

[42] Conoco-Coral, Egyptian General Petroleum Company E. Geologic Map of Egypt. Cairo, Egypt: Conoco-Coral and Egyptian General Petroleum Company (EGPC); 1987.

[43] El Behairy R, El Baroudy A, Ibrahim M, Mohamed E, Rebouh N, Shokr M. Combination of GIS and Multivariate Analysis to Assess the Soil Heavy Metal Contamination in Some Arid Zones. Agronomy. 2022;12(11):2871. doi: 10.3390/agronomy12112871

[44] Shokr MS, Abdellatif MA, El Behairy RA, Abdelhameed HH, El Baroudy AA, Mohamed ES, et al. Assessment of Potential Heavy Metal Contamination Hazards Based on GIS and Multivariate Analysis in Some Mediterranean Zones. Agronomy. 2022;12(12):3220. doi: 10.3390/agronomy12123220

[45] Schumacher BA. Methods for the determination of total organic carbon (TOC) in soils and sediments. US Environmental Protection Agency, Office of Research and Development; 2002.

[46] Hakanson L. An ecological risk index for aquatic pollution control.a sedimentological approach. Water Research. 1980;14(8):975–1001. doi: 10.1016/0043-1354(80)90143-8

[47] Tomlinson DL, Wilson JG, Harris CR, Jeffrey DW. Problems in the assessment of heavy-metal levels in estuaries and the formation of a pollution index. Helgolander Meeresunters. 1980;33(1–4):566–75. doi: 10.1007/bf02414780

[48] Monged MH, Hassan HB, El-Sayed SAJW, Pollution S. Spatial distribution and ecological risk assessment of natural radionuclides and trace elements in agricultural soil of northeastern Nile Valley, Egypt. Air Pollut. 2020;231(7):338.

[49] Sutherland RA. Bed sediment-associated trace metals in an urban stream, Oahu, Hawaii. Environmental Geology. 2000;39(6):611–27. doi: 10.1007/s002540050473

[50] Muller G. Index of geoaccumulation in sediments of the Rhine River. 1969.

[51] Hans Wedepohl K. The composition of the continental crust. Geochimica et Cosmochimica Acta. 1995;59(7):1217–32. doi: 10.1016/0016-7037(95)00038-2

[52] Stoffers P, Glasby G, Wilson C, Davis K, Walter PJN. Heavy metal pollution in Wellington Harbour. NZ J Zool. 1986;20(3):495–512.

[53] Zhao H, Wu Y, Lan X, Yang Y, Wu X, Du L. Comprehensive assessment of harmful heavy metals in contaminated soil in order to score pollution level. Sci Rep. 2022;12(1):3552. doi: 10.1038/s41598-022-07602-9 35241759 PMC8894455

[54] Fei X, Lou Z, Xiao R, Lv X, Christakos G. Contamination and Health Risk Assessment of Heavy Metal Pollution in Soils Developed from Different Soil Parent Materials. Expo Health. 2022;15(2):395–408. doi: 10.1007/s12403-022-00498-w

[55] Ahmad W, Alharthy RD, Zubair M, Ahmed M, Hameed A, Rafique S. Toxic and heavy metals contamination assessment in soil and water to evaluate human health risk. Sci Rep. 2021;11(1):17006. doi: 10.1038/s41598-021-94616-4 34417479 PMC8379239

[56] Wang N, Han J, Wei Y, Li G, Sun Y. Potential Ecological Risk and Health Risk Assessment of Heavy Metals and Metalloid in Soil around Xunyang Mining Areas. Sustainability. 2019;11(18):4828. doi: 10.3390/su11184828

[57] Huang Z, Zheng S, Liu Y, Zhao X, Qiao X, Liu C, et al. Distribution, toxicity load, and risk assessment of dissolved metal in surface and overlying water at the Xiangjiang River in southern China. Sci Rep. 2021;11(1):109. doi: 10.1038/s41598-020-80403-0 33420280 PMC7794442

[58] Mohammadi AA, Zarei A, Esmaeilzadeh M, Taghavi M, Yousefi M, Yousefi Z, et al. Assessment of Heavy Metal Pollution and Human Health Risks Assessment in Soils Around an Industrial Zone in Neyshabur, Iran. Biol Trace Elem Res. 2020;195(1):343–52. doi: 10.1007/s12011-019-01816-1 31317472

[59] Epa USJO, Development W. Exposure factors handbook. 20460. 2011.

[60] Pobi KK, Nayek S, Gope M, Rai AK, Saha R. Sources evaluation, ecological and health risk assessment of potential toxic metals (PTMs) in surface soils of an industrial area, India. Environ Geochem Health. 2020;42(12):4159–80. doi: 10.1007/s10653-020-00517-2 32056062

[61] Bernardo B, Candeias C, Rocha FJG. Soil risk assessment in the surrounding area of hulene-b waste dump, maputo (mozambique). 2022;12(8):290.

[62] Wei M, Pan A, Ma R, Wang H. Distribution characteristics, source analysis and health risk assessment of heavy metals in farmland soil in Shiquan County, Shaanxi Province. Process Safety and Environmental Protection. 2023;171:225–37. doi: 10.1016/j.psep.2022.12.089

[63] She W, Guo L, Gao J, Zhang C, Wu S, Jiao Y, et al. Spatial Distribution of Soil Heavy Metals and Associated Environmental Risks near Major Roads in Southern Tibet, China. Int J Environ Res Public Health. 2022;19(14):8380. doi: 10.3390/ijerph19148380 35886230 PMC9318444

[64] Chu Z, Lin C, Yang K, Cheng H, Gu X, Wang B, et al. Lability, bioaccessibility, and ecological and health risks of anthropogenic toxic heavy metals in the arid calcareous soil around a nonferrous metal smelting area. Chemosphere. 2022;307(Pt 4):136200. doi: 10.1016/j.chemosphere.2022.136200 36030943

[65] Yang Y, Yang X, He M, Christakos G. Beyond mere pollution source identification: Determination of land covers emitting soil heavy metals by combining PCA/APCS, GeoDetector and GIS analysis. CATENA. 2020;185:104297. doi: 10.1016/j.catena.2019.104297

[66] González-Valoys AC, Esbrí JM, Campos JA, Arrocha J, García-Noguero EM, Monteza-Destro T, et al. Ecological and Health Risk Assessments of an Abandoned Gold Mine (Remance, Panama): Complex Scenarios Need a Combination of Indices. Int J Environ Res Public Health. 2021;18(17):9369. doi: 10.3390/ijerph18179369 34501959 PMC8431601

[67] Topaldemir H, Taş B, Yüksel B, Ustaoğlu F. Potentially hazardous elements in sediments and Ceratophyllum demersum: an ecotoxicological risk assessment in Miliç Wetland, Samsun, Türkiye. Environ Sci Pollut Res Int. 2023;30(10):26397–416. doi: 10.1007/s11356-022-23937-2 36367653

[68] Zhou Y, Niu L, Liu K, Yin S, Liu W. Arsenic in agricultural soils across China: Distribution pattern, accumulation trend, influencing factors, and risk assessment. Sci Total Environ. 2018;616–617:156–63. doi: 10.1016/j.scitotenv.2017.10.232 29112838

[69] Howladar MF, Hossain MN, Anju KA, Das DJ. Ecological and health risk assessment of trace metals in water collected from Haripur gas blowout area of Bangladesh. Ecotoxicol Environ Saf. 2021;11(1):15573. 10.1038/s41598-021-94830-0 PMC8329277 34341388

[70] Mazhar M, Ahmed S, Husain A, Uddin RJP. Monitoring of trihalomethanes and its cancer risk assessment in drinking water of Delhi City, India. RJP. 2022;8(3):830–43.

[71] Zhang Z, Zhang N, Li H, Lu Y, Wang Q, Yang Z. Risk assessment, spatial distribution, and source identification of heavy metal(loid)s in paddy soils along the Zijiang River basin, in Hunan Province, China. J Soils Sediments. 2019;19(12):4042–51. doi: 10.1007/s11368-019-02352-0

[72] Einar K, Ibrahim M, Abdelsamie E, Shokr M. Modeling land degradation in drylands of the Nile Delta using remote sensing and GIS. Egyptian Journal of Soil Science. 2025;65(2).

[73] Health UDo, Health HSJAUDo, Services H. Agency for Toxic Substances and Disease Registry: Toxicological profile for Lead (update) PB/99/166704. 1999.

[74] Alloway B, Steinnes E, McLaughlin M, Singh B. Cadmium in soils and plants. Dordrecht: Kluwer Academic Publisher; 1999.

[75] Forstiner A, Wittman AJBH. Metal pollution in aquatic environment. Germany; 1983.

[76] Nazzal Y, Rosen MA, Al-Rawabdeh AM. Assessment of metal pollution in urban road dusts from selected highways of the Greater Toronto Area in Canada. Environ Monit Assess. 2013;185(2):1847–58. doi: 10.1007/s10661-012-2672-3 22644122

[77] Parvez MS, Nawshin S, Sultana S, Hossain MS, Rashid Khan MH, Habib MA, et al. Evaluation of Heavy Metal Contamination in Soil Samples around Rampal, Bangladesh. ACS Omega. 2023;8(18):15990–9. doi: 10.1021/acsomega.2c07681 37179636 PMC10173447

[78] Ogundele D, Adio A, Oludele OJ. Heavy metal concentrations in plants and soil along heavy traffic roads in North Central Nigeria. Journal of Toxicology. 2015;5(6):1.

[79] ATSDR (United States Agency for Toxic Substances and Disease Registry). oxico logical Profile for Cobalt. T US Department of Health and Human Services, US Department of Health and Human Services. 2004. p 486.

[80] Fishel FMJE. Pesticide Toxicity Profile: Copper-based Pesticides: PI-66/PI103. 11. 2005.

[81] Luo D, Zheng H, Chen Y, Wang G, Fenghua DJ. Transfer characteristics of cobalt from soil to crops in the suburban areas of Fujian Province, southeast China. J Environ Manage. 2010;91(11):2248–53. 20615604 10.1016/j.jenvman.2010.06.001

[82] DEA. National environmental management, waste act, 2008; national norms Standards for the remediation of contaminated land and soil quality in the republic of South Africa. Pretoria, South Africa: Government Gazette 36447; 2013.

[83] Taylor SR, McLennan SMJ. The geochemical evolution of the continental crust. Geochimica et Cosmochimica Acta. 1995;33(2):241–65.

[84] Bradl H. Heavy metals in the environment: origin, interaction and remediation. Elsevier; 2005.

[85] Abuzaid AS, Jahin HS, Shokr MS, El Baroudy AA, Mohamed ES, Rebouh NY, et al. A Novel Regional-Scale Assessment of Soil Metal Pollution in Arid Agroecosystems. Agronomy. 2023;13(1):161. doi: 10.3390/agronomy13010161

[86] Kelepertzis E. Accumulation of heavy metals in agricultural soils of Mediterranean: Insights from Argolida basin, Peloponnese, Greece. Geoderma. 2014;221–222:82–90. doi: 10.1016/j.geoderma.2014.01.007

[87] Mohamed ES, Jalhoum ME, Hendawy E, El-Adly AM, Nawar S, Rebouh NY. Geospatial evaluation and bio-remediation of heavy metal-contaminated soils in arid zones. 2024;12:1381409.

[88] Rodríguez Martín JA, Ramos-Miras JJ, Boluda R, Gil C. Spatial relations of heavy metals in arable and greenhouse soils of a Mediterranean environment region (Spain). Geoderma. 2013;200–201:180–8. doi: 10.1016/j.geoderma.2013.02.014

[89] Xia X, Chen X, Liu R, Liu H. Heavy metals in urban soils with various types of land use in Beijing, China. J Hazard Mater. 2011;186(2–3):2043–50. doi: 10.1016/j.jhazmat.2010.12.104 21242029

[90] Alharbi T, El-Sorogy AS, Al-Kahtany K, Al-Hashim MH. Source of contamination and assessment of potential health risks of potentially toxic metal (loid) s in agricultural soil from Al Lith, Saudi Arabia. JOG. 2025;17(1):20250782.

[91] Alzahrani H, El-Sorogy AS, Okok A, Shokr MS. GIS- and Multivariate-Based Approaches for Assessing Potential Environmental Hazards in Some Areas of Southwestern Saudi Arabia. Toxics. 2024;12(8):569. doi: 10.3390/toxics12080569 39195671 PMC11359128

[92] Kumar V, Bhatti SS, Nagpal AK. Assessment of Metal(loid) Contamination and Genotoxic Potential of Agricultural Soils. Arch Environ Contam Toxicol. 2021;81(2):272–84. doi: 10.1007/s00244-021-00874-8 34272567

[93] Guan Y, Shao C, Ju M. Heavy metal contamination assessment and partition for industrial and mining gathering areas. Int J Environ Res Public Health. 2014;11(7):7286–303. doi: 10.3390/ijerph110707286 25032743 PMC4113876

[94] Xia F, Zhang C, Qu L, Song Q, Ji X, Mei K, et al. A comprehensive analysis and source apportionment of metals in riverine sediments of a rural-urban watershed. J Hazard Mater. 2020;381:121230. doi: 10.1016/j.jhazmat.2019.121230 31563037

[95] Mendoza EO, Custodio M, Ascensión J, Bastos MC. Heavy metals in soils from high andean zones and potential ecological risk assessment in Peru’s central andes. Journal of Environmental Engineering. 2020;21(8).

[96] Cai L, Xu Z, Bao P, He M, Dou L, Chen L, et al. Multivariate and geostatistical analyses of the spatial distribution and source of arsenic and heavy metals in the agricultural soils in Shunde, Southeast China. Journal of Geochemical Exploration. 2015;148:189–95. doi: 10.1016/j.gexplo.2014.09.010

[97] Tóth G, Hermann T, Da Silva MR, Montanarella L. Heavy metals in agricultural soils of the European Union with implications for food safety. Environ Int. 2016;88:299–309. 26851498 10.1016/j.envint.2015.12.017

[98] Cheng Z, Paltseva A, Li I, Morin T, Huot H, Egendorf S, et al. Trace Metal Contamination in New York City Garden Soils. Soil Science. 2015;180(4/5):167–74. doi: 10.1097/ss.0000000000000126

[99] Ma L, Sun J, Yang Z, Wang LJ. Heavy metal contamination of agricultural soils affected by mining activities around the Ganxi River in Chenzhou, Southern China. Environmental Monitoring and Assessment. 2015;187:1–9. 26547321 10.1007/s10661-015-4966-8

[100] Yaylalı-Abanuz GJMJ. Heavy metal contamination of surface soil around Gebze industrial area, Turkey. GJMJ. 2011;99(1):82–92.

[101] Kabata-Pendias A. Trace elements in soils and plants. CRC Press; 2000.

[102] Pierart A, Shahid M, Séjalon-Delmas N, Dumat C. Antimony bioavailability: knowledge and research perspectives for sustainable agricultures. J Hazard Mater. 2015;289:219–34. doi: 10.1016/j.jhazmat.2015.02.011 25726907

[103] Abuzaid AS, Jahin HS. Profile Distribution and Source Identification of Potentially Toxic Elements in North Nile Delta, Egypt. Soil and Sediment Contamination: An International Journal. 2019;28(6):582–600. doi: 10.1080/15320383.2019.1637818

[104] Emam WW, Soliman KMJ, Assessment R. Geospatial analysis, source identification, contamination status, ecological and health risk assessment of heavy metals in agricultural soils from Qallin city, Egypt. Sustainability and Environmental Research. 2022;36(9):2437–59.

[105] Shaheen SM, Antoniadis V, Kwon E, Song H, Wang S-L, Hseu Z-Y, et al. Soil contamination by potentially toxic elements and the associated human health risk in geo- and anthropogenic contaminated soils: A case study from the temperate region (Germany) and the arid region (Egypt). Environ Pollut. 2020;262:114312. doi: 10.1016/j.envpol.2020.114312 32193081

[106] Alloway BJ. Heavy metals in soils: trace metals and metalloids in soils and their bioavailability: Springer Science & Business Media; 2012.

[107] Garzanti E, Andò S, Limonta M, Fielding L, Najman Y. Diagenetic control on mineralogical suites in sand, silt, and mud (Cenozoic Nile Delta): Implications for provenance reconstructions. Earth-Science Reviews. 2018;185:122–39. doi: 10.1016/j.earscirev.2018.05.010

[108] Yang Y, Christakos G, Guo M, Xiao L, Huang W. Space-time quantitative source apportionment of soil heavy metal concentration increments. Environ Pollut. 2017;223:560–6. doi: 10.1016/j.envpol.2017.01.058 28131479

[109] Zhao R, Guan Q, Luo H, Lin J, Yang L, Wang F, et al. Fuzzy synthetic evaluation and health risk assessment quantification of heavy metals in Zhangye agricultural soil from the perspective of sources. Sci Total Environ. 2019;697:134126. doi: 10.1016/j.scitotenv.2019.134126 31491630

[110] Taghavi M, Bakhshi K, Zarei A, Hoseinzadeh E, Gholizadeh A. Soil pollution indices and health risk assessment of metal(loid)s in the agricultural soil of pistachio orchards. Sci Rep. 2024;14(1):8971. doi: 10.1038/s41598-024-59450-4 38637594 PMC11026477

[111] Ghani J, Nawab J, Faiq ME, Ullah S, Alam A, Ahmad I, et al. Multi-geostatistical analyses of the spatial distribution and source apportionment of potentially toxic elements in urban children’s park soils in Pakistan: A risk assessment study. Environ Pollut. 2022;311:119961. doi: 10.1016/j.envpol.2022.119961 35977638

[112] Ali L, Rashid A, Khattak SA, Zeb M, Jehan S. Geochemical control of potential toxic elements (PTEs), associated risk exposure and source apportionment of agricultural soil in Southern Chitral, Pakistan. Microchemical Journal. 2019;147:516–23. doi: 10.1016/j.microc.2019.03.034

[113] Caravanos J, Weiss AL, Blaise MJ, Jaeger RJ. A survey of spatially distributed exterior dust lead loadings in New York City. Environ Res. 2006;100(2):165–72. doi: 10.1016/j.envres.2005.05.001 16005864

