# Supplementary material for: Health risk assessment of heavy metals in arid soils of the Nile Delta, Egypt using GIS and multivariate statistical analyses
Source: PLoS One. 2025 Nov 10;20(11):e0335394. doi: 10.1371/journal.pone.0335394 (PMC12599927; doi:10.1371/journal.pone.0335394)
Supplement: S1 File — (DOCX) [file pone.0335394.s001.docx]

Table S1 LOQ and LOD of studied elements

| Elements | Measuring unit | LOQ | LOD |
| --- | --- | --- | --- |
| As | mg Kg^-1^ | 2 | 6.66 |
| Cd |  | 0.1 | 0.03 |
| Co |  | 1 | 0.30 |
| Cu |  | 1 | 0.30 |
| Fe |  | 100 | 30.30 |
| Ni |  | 1 | 0.30 |
| Mn |  | 5 | 1.51 |
| Pb |  | 2 | 0.60 |
| Zn |  | 2 | 0.60 |

Table S2 the recovery % of studied HMs

| Elements | As | Co | Cr | Cu | Fe | Ni | Pb | V | Zn |
| --- | --- | --- | --- | --- | --- | --- | --- | --- | --- |
| Measured values | 3.5 | 2 | 12 | 6 | 6400 | 10 | 9 | 14 | 40 |
| Certificate values | 3.5 | 2 | 12 | 6 | 6400 | 10.5 | 9 | 14 | 40 |
| Recovery % | 100 | 100 | 100 | 100 | 100 | 95.23 | 100 | 100 | 100 |

Table S3 Enrichment factor, Igeo, and CF indices thresholds and classes

| EF value | Contamination level |
| --- | --- |
| <1 | Natural origin |
| 1-2 | Low enrichment |
| 2-5 | Moderate enrichment |
| 5-20 | Significant enrichment |
| 20-40 | Very high enrichment |
| >40 | Severe enrichment |
| I_geo_ index | Interpretation |
| ≤0 | Unpolluted |
| 0-1 | Unpolluted to moderately polluted |
| 1-2 | Moderately polluted |
| 2-3 | Moderately to strongly polluted |
| 3-4 | Strongly polluted |
| 4-5 | Strongly to extremely polluted |
| >5 | Extremely polluted |
| CF | Classes |
| < 1 | Low contamination |
| 1 – 3 | Moderate contamination |
| 3 – 6 | Considerable contamination |
| ˃ 6 | Very high contamination |

**Table S4.**Er and RI classes within study area

| **Index** | **Value** | **Category** |
| --- | --- | --- |
| **Er** | < 40 | Low potential ecological risk |
|  | 40 ≤ Er < 80 | Moderate potential ecological risk |
|  | 80 ≤ Er < 160 | Considerable potential ecological risk |
|  | 160 ≤ Er < 320 | High potential ecological risk |
|  | Er ≥ 320 | Very high ecological risk |
| **RI** | < 150 | Low ecological risk |
|  | 150 ≤ RI < 300 | Moderate ecological risk |
|  | 300 ≤ RI < 600 | Considerable ecological risk |
|  | RI > 600 | Very high ecological risk |

**Table S5.** Input parameters indicate the average daily intake of trace elements through various exposure paths.

| Parameters | Symbols | Value | | Units | Reference |
| --- | --- | --- | --- | --- | --- |
|  |  | Adults | Children |  |  |
| Ingestion rate | R_ing_ | 100 | 200 | mg/day | [1-3] |
| Inhalation rate | *C_inh_* | 7.6 | 20 | mg/day |  |
| Exposure frequency | *EF* | 350 | 350 | Days/year |  |
| Exposure duration | *ED* | 24 | 6 | Years |  |
| skin area | *SA* | 5700 | 2800 | cm^2^ |  |
| Adherence factor of soil | *AF* | 0.07 | 0.2 | mg/m^2^·day |  |
| Dermal absorption factor | *ABS* | 0.001 | 0.001 | - |  |
| Particle emission factor | *PEF* | 1.36E+09 | 1.36E+09 | m^3^/kg |  |
| Average timing | *AT* | 8760 | 2190 | Days |  |
| Average bodyweight | *BW* | 70 | 15 | Kg |  |

**Table S6.** The reference dose (Rfd) and slope factor (SF) values that are used to quantify the risk of HMs.

| Metals | *Rfd* (mg kg^-1^ day^-1^) | | | Reference | *CSF* **(**mg kg^-1^ day^-1^) | | | Reference |
| --- | --- | --- | --- | --- | --- | --- | --- | --- |
|  | Ingestion | Dermal | Inhalation |  | Ingestion | Dermal | Inhalation |  |
| As | 3.00E-04 | 3.01E-04 | 1.23E-04 | [1, 4] | 1.50E+00 | 3.66 | 1.51E+01 | [5] |
| Cd | 1.00E-03 | 1.00E-03 | 1.00E-03 |  | 5.10E-01 | 2.00E+01 | 6.30E+00 |  |
| Co | 3.00E-03 | 3.00E-03 | 3.00E-03 |  | ـــــــ | ـــــــ | 9.8 |  |
| Cu | 4.00E-02 | 4.00E-02 | 4.00E-02 |  | 1.70E+00 | 1.70E+00 | ـــــــ |  |
| Fe | 7.00E-01 | 7.00E-01 | 7.00E-01 |  | ـــــــ | 2.00E+01 | ـــــــ |  |
| Mn | 1.40E-02 | 1.40E-02 | 1.40E-02 |  | ـــــــ | ـــــــ | ـــــــ |  |
| Ni | 2.00E-02 | 2.00E-02 | 2.00E-02 |  | 1.7 | 42.5 | 8.40E-01 |  |
| Pb | 3.50E-03 | 3.50E-03 | 3.50E-03 |  | 8.50E-03 | 8.5 E -03 | 0.042 |  |
| Zn | 3.00E-01 | 3.00E-01 | 3.00E-01 |  | ـــــــ | ـــــــ | ـــــــ |  |

1. Epa USJOor, Development W, DC. Exposure factors handbook. 2011;20460:2-6.

2. Karimi A, Naghizadeh A, Biglari H, Peirovi R, Ghasemi A, Zarei AJES, et al. Assessment of human health risks and pollution index for heavy metals in farmlands irrigated by effluents of stabilization ponds. 2020;27:10317-27.

3. Raj D, Maiti SKJEM, Assessment. Risk assessment of potentially toxic elements in soils and vegetables around coal-fired thermal power plant: A case study of Dhanbad, India. 2020;192(11):699.

4. Adimalla NJEG, Health. Heavy metals contamination in urban surface soils of Medak province, India, and its risk assessment and spatial distribution. 2020;42(1):59-75.

5. Miletić A, Lučić M, Onjia AJM. Exposure factors in health risk assessment of heavy metal (loid) s in soil and sediment. 2023;13(7):1266.
